# History shapes regulatory and evolutionary responses to tigecycline in two reference strains of Acinetobacter baumannii

**DOI:** 10.1099/mic.0.001570

**Published:** 2025-06-09

**Authors:** Alecia B. Rokes, Alfonso Santos-Lopez, Vaughn S. Cooper

**Affiliations:** 1 Department of Microbiology and Molecular Genetics, University of Pittsburgh, Pittsburgh, PA, USA; 2 Center for Evolutionary Biology and Medicine, University of Pittsburgh, Pittsburgh, PA, USA; 3 Department of Molecular Biology, Universidad Autónoma de Madrid, Madrid, Spain; 4 Consorcio de Investigación Biomédica en Red de Epidemiología y Salud Pública (CIBERESP), Madrid, Spain

**Keywords:** *Acinetobacter baumannii*, antimicrobial resistance, evolutionary history, experimental evolution, transcriptomics

## Abstract

Evolutionary history encompasses both genetic and phenotypic bacterial differences; however, the extent to which this history influences drug response and antimicrobial resistance (AMR) adaptation remains unclear. Historical contingencies arise when elements from an organism’s past leave lasting effects on the genome, altering the paths available for adaptation. Here, we compare two diverging reference strains of *Acinetobacter baumannii*, representative of archaic and contemporary infections, to study the impact of deep historical differences shaped by decades of adaptation in varying antibiotic and host pressures. We evaluated these effects by comparing immediate and adaptive responses to the last-resort antibiotic, tigecycline (TGC). The strains demonstrated divergent transcriptional responses, suggesting that baseline transcript levels may dictate global responses to antibiotics. Experimental evolution in TGC revealed clear differences in population dynamics, with hard sweeps in populations founded by one strain and no mutations reaching fixation in the other strain. AMR was acquired through predictable mechanisms of increased efflux and drug target modification; however, efflux targets were dictated by strain background. Genetic adaptation may outweigh historic differences in transcriptional networks, as evolved populations no longer show transcriptomic signatures of drug response. Importantly, fitness–resistance trade-offs were only observed in lineages evolved from the archaic strain, while the contemporary reference isolate suffered no fitness defects. This suggests that decades of adaptation to antibiotics resulted in pre-existing compensatory mechanisms in the more contemporary isolate, an important example of a beneficial effect of historical contingencies.

**Impact Statement**

*Acinetobacter baumannii* is a high-priority pathogen often causing multidrug-resistant nosocomial infections. Many healthcare systems experience clonal outbreaks of *A. baumannii* infections, yet treatment strategies are often strain-agnostic, ignoring the importance of strain differences. We show that historical differences between two reference strains, one isolated prior to widespread antibiotic use and the other following decades of selection to clinical conditions, dictate transcriptional patterns and response to a last-resort antibiotic and influence the genetic and phenotypic routes of resistance adaptation. While our study focuses on two reference strains of *A. baumannii*, these findings can be more broadly applicable to other pathogenic organisms in which a better understanding of the forces influencing resistance adaptation is essential for combating the antimicrobial resistance crisis.

## Data Summary

All genomic sequencing data have been uploaded to the NCBI BioProject ID PRJNA1214285. The full list of accession numbers for BioSamples within this BioProject can be found in the supplemental material, available with the online version of this article. The annotated reference genomes, supplemental data and supplemental tables are available at https://github.com/vscooper/tigecycline.

## Introduction

Evolution is an inescapable process, as the worsening antimicrobial resistance (AMR) crisis demonstrates [1]. The AMR crisis will be less daunting if we are better able to understand, predict and direct the evolutionary outcomes of antibiotic treatment [2,3]. With better resolution of factors affecting the routes to resistance, we may be able to work toward directing evolution to avenues with exploitable resistance trade-offs [4,5]. However, genotypic and phenotypic routes to resistance, as well as the resulting fitness trade-offs, may be highly influenced by strain history, decreasing the range and applicability of evolutionary strategies to counteract the evolution of AMR [6,7].

There are three primary forces, often intertwined, that influence evolution: history, chance and selection [5,8, 9]. Natural selection causes the most fit alleles in a population to rise in frequency, while less fit mutants are lost from the population. Selection needs heritable phenotypic variance to act upon, which is produced by mutations arising randomly, a factor of chance. Chance events also occur in the demography of microbial populations, such as population bottlenecks arising during infections [10]. The outcome of a mutation is also affected by the genetic background in which it arises [11,12], combining chance and the final force of evolutionary history, which can influence adaptation in diverse ways but remains understudied.

Evolutionary history encompasses the genetic and phenotypic remnants from a bacterium’s past, including biotic and abiotic factors, such as interactions with other cells and previous environmental exposures like antibiotics, respectively [13]. The phylogenetic origin of the bacterium is also a major component of evolutionary history. These differences can impose historical contingencies through which pre-existing elements in the genome, transcriptome or phenome alter or constrain evolutionary paths available for adaptation [13,14]. History may dictate the type, identity, rate and order of mutations acquired [15,18]. Additionally, history likely influences fitness and phenotype associated with a mutation, thereby dictating evolutionary trajectories and outcomes [19,21]. This study seeks to understand how historical contingencies influence evolutionary trajectories under the strong selective pressure of antibiotic treatment [22].

Contingencies imposed by history exacerbate our inability to accurately predict bacterial responses to antibiotics and AMR evolution. *Acinetobacter baumannii* is a nosocomial pathogen of increasing concern due to limited and often ineffective treatment options [23]. With time and increased antibiotic use, strains of *A. baumannii* have acquired mutations and mobile genetic elements, on top of many conserved genes, that endow desiccation resistance, stress tolerance and drug resistance [24,25]. Such historical differences could cause archaic strains, which are often represented by laboratory reference strains, to respond and evolve differently than contemporary infectious strains [26,27]. Importantly, hospitals typically experience clonal outbreaks of *A. baumannii* caused by closely related isolates of the same sequence type [28,29]. Across hospital systems, *A. baumannii* infections exhibit high diversity [30,31], but treatment strategies are often based solely on rapid resistance profiling. Treatments often ignore the importance of genetic background and evolutionary history, leading to potential differences in bacterial response and treatment success [32,33]. A better understanding of how various strains respond and adapt to antibiotics would result in improved patient outcomes.

The widespread use of antibiotics in clinical practice has dramatically affected pathogen evolution [7,34]. Comparing strains isolated before and after the clinical introduction of antibiotics provides an excellent framework to study how deep historical differences influence the evolution of resistance [35]. To this end, we used two reference strains of *A. baumannii,* one that is widely used but highly diverged from current infections and one that is better representative of current multidrug-resistant (MDR) clinical infections, having been isolated much more recently. Directly comparing these strains enables assessment of how deep historical differences impact resistance evolvability in two widely utilized strains.

Resistance adaptation can be investigated at multiple levels: from the narrowest, the site-specificity of individual mutations, to more systematic resistance pathways, and even more broadly, at the level of population-wide responses and adaptive dynamics [36,37]. This study looks within and across these levels to determine the areas in which evolutionary history is most influential. We find that deep history influences the basal transcriptome, driving differences in the response to tigecycline (TGC). The strains also differed in their evolved responses to prolonged TGC stress at both narrow molecular-genetic and broader population-genetic levels, despite superficial similarities. This work highlights the importance of evolutionary history in the response and adaptation of a high-priority pathogen to a last-resort antibiotic, showing that strain differences can reduce the predictability of treatment outcomes.

## Methods

### Bacterial strains and growth conditions

Laboratory reference strain ATCC 17978UN [38,39] and clinical reference strain AB5075-UW [40,41] were used throughout the study and grown under the same conditions. Unless otherwise stated, bacterial cultures were grown in 5 ml M9Plus media, as previously described [42]. Briefly, M9Plus media is a salts-buffered media with glucose (11.1 mM) as the primary carbon source. It contains 0.1 mM CaCl_2_, 1 mM MgSO_4_, 42.2 mM Na_2_HPO_4_, 22 mM KH2PO_4_, 21.7 mM NaCl and 18.7 mM NH_4_Cl and is supplemented with 20 ml l^−1^ MEM essential amino acids (Gibco 11130051), 10 ml l^−1^ MEM nonessential amino acids (Gibco 11140050) and 1 ml each of trace mineral solutions A, B and C (Corning 25021–3 Cl). Cultures were incubated at 37 °C with shaking or on a roller-drum at ~250 r.p.m.

### Genomic comparison of the two strains

Genomes were annotated with bakta (v1.6.1, database v4.0) [43] and input to Panaroo (v1.3) in the sensitive clean mode to obtain a gene presence/absence list that included plasmid-encoded genes [44]. We assessed pairwise nucleotide similarity of the two strains with pyANI (v0.2.12) [45]. Multilocus sequence types were confirmed using mlst (v2.11; https://github.com/tseemann/mlst) [46]. Elements associated with resistance, including known SNPs, were detected with AMRfinderPlus (version 3.11.26 with database version 2023-11-15) [47]. Two-proportion z-tests on two-tailed hypotheses were used to determine if gene content was significantly different in the two strains, accounting for the difference in genome size (Table S1, available in the online Supplementary Material). The annotated reference genomes used in this study can be found at https://github.com/vscooper/tigecycline.

### Measuring resistance

Broad resistance profiles of the two strains were determined using Sensitire plates (Thermo Fisher, GN3F) following the manufacturer’s protocols. MIC assays were performed to measure susceptibility levels more accurately to TGC (Sigma 220620-09-7) following the modified CLSI methods [9,48]. More detailed methods can be found in the supplement.

### RNA extraction and purification

Cultures for transcriptomic analysis of the ancestor strains were seeded from individual colonies in biological triplicate into 5 ml M9Plus media. Population cultures were started directly from the freezer stock into 5 ml M9Plus to avoid unnecessary outgrowths. Dilutions of the cultures into M9Plus with or without TGC treatment at 0.06 µg ml^−1^ were grown to mid-late exponential phase for RNA extraction. RNA was extracted following a modified TRIzol (Invitrogen Cat. No. 15596026) protocol, purified using the Invitrogen PureLink^®^ RNA Mini Kit (Thermo Cat. No. 12183025) and treated twice with DNase. See supplemental methods for more details.

### RNA sequencing and analysis

Full details for RNA sequencing and analysis can be found in the supplemental methods. Briefly, once RNA was purified and satisfied quality control measures, we performed ribosomal RNA depletion using the RiBO-COP rRNA Depletion Kit for Gram-Negative Bacteria (Lexogen Cat. No. 126.96). Library generation was done using Lexogen RNA sequencing kits, following the manufacturer’s protocols. Sequencing was performed in-house on an Illumina NextSeq550 with the corresponding High-Output Kit v2.5 75 cycles (Illumina 20024906). Raw reads are uploaded to the NCBI BioProject ID PRJNA1214285. Reads were assessed for quality, processed and aligned to the reference genomes using kallisto [49]. This analysis provided the gene abundance metrics, measured in transcripts per million transcripts, which normalize by both gene length and sequencing depth. Differential expression analysis was done with DESeq2 [50]. Unless otherwise stated, cut-offs for significance were set to a false discovery rate-adjusted *P*-value<0.05 and a magnitude of log_2_ fold change >1. RNA sequencing was performed on three biological replicates for each sample by treatment combination, except for the removal of two replicates due to low sequencing coverage or outlier status (17978UN population one untreated replicate A and AB5075-UW population two untreated replicate C), and averages of the replicates are presented.

### Experimental evolution with TGC

Our experimental design was adapted from previous studies [42,51]. Lineages were inoculated from an overnight culture with a 1:100 dilution into fresh M9Plus with or without TGC, resulting in ~6.6 generations per day [52]. The experiment was designed such that the populations were not mutation-limited (see supplemental methods). In the TGC-treated condition, the TGC level in the media was tailored to the initial susceptibility level of each strain, starting at 0.5× the ancestral MIC (subinhibitory). Every 3 days, the concentration of TGC in the media was doubled (Fig. S5). We propagated the lineages for 12 days, with the final TGC media concentration at 4× the ancestral MIC, which crossed the clinical breakpoint for resistance in both strains. Populations were periodically sampled and frozen in 9% DMSO at −80 °C, as well as pelleted and frozen at −20 °C for DNA extraction. Following standard practice in the field, phenotypic and genotypic analysis were performed on three evolutionary replicates per strain per condition [5,9, 53].

### Whole-population, whole-genome sequencing and analysis

DNA was extracted from frozen cell pellets using the DNeasy blood and tissue kit for the QIAcube (QIAGEN, Hilden, Germany) with a 10-min elution into nuclease-free water. Libraries were prepared in-house as previously described [54] or using the plexWell^TM^ kit following the manufacturer’s directions (SeqWell PW096). Libraries were sequenced using an Illumina NextSeq550 sequencer with a 300-cycle mid-output kit (Illumina 20024905). Raw reads are uploaded to the NCBI BioProject ID PRJNA1214285. Reads were demultiplexed, trimmed and quality checked, as described in the supplemental methods for the RNAseq reads. Breseq (v0.35.0) was used for read mapping and variant calling [55] with subsequent filtering following previously published rationale [51] with special attention to new junction calls. Filtering, consolidating for allele frequencies and plotting were done in RStudio (R v4.2.1) with the packages ggplot2 (v3.4.2; https://CRAN.R-project.org/package=ggplot2) and tidyr (v1.3.0; https://CRAN.R-project.org/package=tidyr).

### Growth curves as a measure of fitness

We use bacterial growth curves to measure absolute fitness of ancestral clonal samples as well as to measure aggregate absolute fitness of evolved populations [56]. Growth curves were seeded to mimic the transfers of the evolution experiment. A large sample of the frozen population (or ancestor) stock was inoculated, in biological triplicate, into M9Plus media and grown for 24 h. Overnight cultures were then diluted 1:100 into fresh media, with or without TGC, and OD_600_ measured every 10 min for 24 h. Fitness was measured, in technical triplicate, in plain M9Plus as well as in M9Plus containing 0.06 µg ml^−1^ TGC. We use the area under the curve (AUC) to measure the fitness of populations normalized by the AUC of their respective ancestor (AUC_evolved population_/AUC_ancestor average_). Analyses were done in RStudio (R v4.2.1) utilizing previously published pipelines [57] (https://github.com/mjfritz/Growth_Curves_in_R).

### Measurements of efflux expression and activity

Efflux activity of late exponential phase cultures was assessed using the ethidium bromide efflux activity assay. Fluorescence intensity was measured at 3 min post-addition of ethidium bromide to washed cells with an excitation of 530 nm and emission of 600 nm. Ancestral efflux activity and expression were measured in M9Plus alone or with the addition of 0.06 µg ml^−1^ TGC and only in plain M9Plus for the evolved populations. Quantitative reverse transcription PCR was used to measure expression of efflux pump components. RNA extraction was done as previously described (supplemental methods). The Power SYBR Green RNA-to-Ct 1-Step Kit (Applied Biosystems) was used for cDNA synthesis and quantitative PCR with custom primers (Table S4). Data were analysed for ΔCt and ΔΔCt, normalized by rpoB and ancestor, respectively, within the strain background. Expression values are presented as 2^-normalized expression^, such that higher values indicate increased expression. We used the main efflux pump gene (adeB, G and J) as a representative for pump expression [58,59]. More information on assays of efflux activity and expression can be found in the supplemental methods.

## Results

### Genomic and phenotypic classifications of the strains

Previous studies of historical differences in strain response to antibiotics have used strains separated by relatively few mutations [9]. Here, we study the effects of deeper history on the evolution of AMR in two different *A. baumannii* reference strains isolated nearly 60 years apart. The first strain, 17978UN, is a variant of the commonly used laboratory reference strain ATCC 17978 [38]. This strain was isolated from a child with meningitis in 1951 in the early stages of the introduction of antibiotics in clinical practice [39]. The existence of multiple variants of ATCC 17978 exemplifies the impact of unintended laboratory propagation on genetic adaptation within a reference strain [38]. In contrast, the second strain, AB5075-UW, is a variant of AB5075, which was isolated in 2008 from an MDR infection representative of the current global clinical burden of *A. baumannii* [40,41]. Therefore, the evolutionary histories of both strains have likely been shaped by different exposures to antibiotics, hospitals and the human host. In many ways, 17978UN represents archaic *A. baumannii* infections in comparison to contemporary MDR infections represented by AB5075-UW.

As much is already known about these strains, both individually and in comparison to each other [30,60, 61], we performed a broad-level characterization of genomic and phenotypic differences between the two strains to evaluate their historical distinctions. 17978UN is a member of sequence type ST437, whereas AB5075-UW is assigned to ST1, part of the clinically dominant clonal complex 1 [40]. Their genomes share an average nucleotide identity of 97.5% and 3,265 homologous core genes. 17978UN contains an additional 694 accessory genes, whereas AB5075-UW encodes 751 accessory genes (Data S1), with no significant differences in gene content grouped by Clusters of Orthologous Genes categories (Table S1). However, as expected by its lineage and history of antibiotic exposure, AB5075-UW has significantly more resistance-associated elements than 17978UN (Table S1) and is more resistant to a variety of antibiotics (Table S2). Notably, both strains were susceptible to at least two different classes of antibiotics.

These significant differences in genome content and existing AMR led to the hypothesis that the strains would respond differently to a contemporary, clinically relevant antibiotic. Tigecycline (TGC) is a translation-inhibiting antimicrobial compound that was approved for clinical use in 2005 [62]. Resistance to TGC in clinical isolates of *A. baumannii* is typically attributed to overexpression of efflux pumps or ribosomal modifications [63,64]. Both strains are initially susceptible to TGC; the MIC of TGC differed twofold between the strains (17978UN MIC_TGC_=0.125 µg ml^−1^, AB5075-UW MIC_TGC_=0.50 µg ml^−1^). Further, 0.06 µg ml^−1^ TGC imposed ~75% and 60% fitness defects in 17978UN and AB5075-UW, respectively (Fig. S1). While this level of growth inhibition is comparable, we acknowledge the slight advantage AB5075-UW may have at this drug level as a confounding factor that is a historically contingent trait likely due to this strain’s more recent clinical isolation. Challenge by the same concentration of TGC enables the study and comparison of the influence of deep evolutionary history on both transcriptional response and subsequent evolutionary adaptation.

### Strain-dependent transcriptional response acts on historically differentially utilized genes

**Fig. 1.**
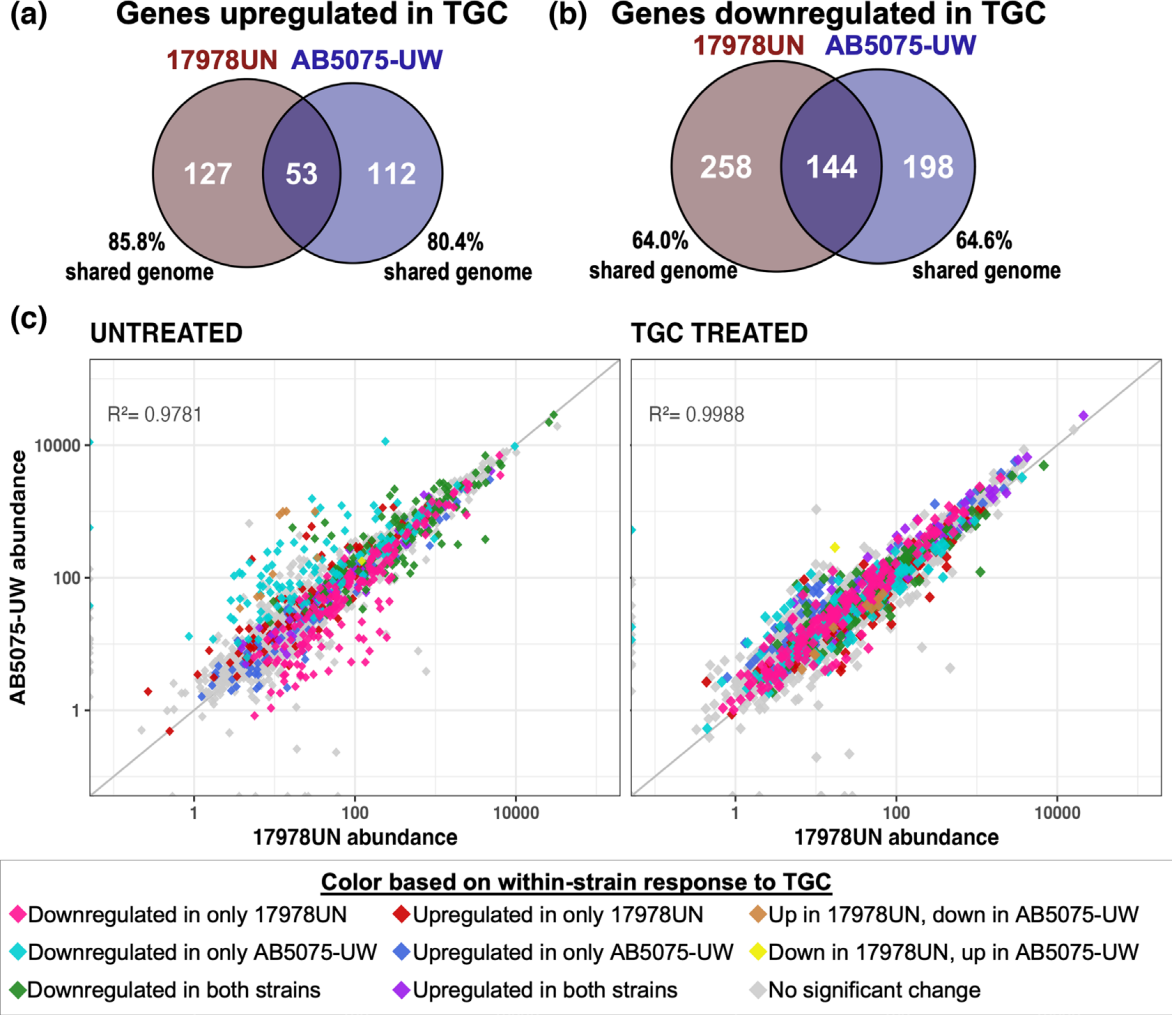
Transcriptional response to TGC is highly strain dependent and results in a shift towards a more conserved transcriptional state. Counts of differentially expressed genes, upregulated (**a**) or downregulated (**b**), in each strain in response to TGC treatment. Overlapping regions represent genes that were similarly differentially expressed in both strains. The percentage of genes that were significantly differentially expressed in one strain but are still encoded by both strains (shared genome) is indicated, showing minimal involvement of accessory genes. (**c**) Each point represents the abundance of one gene in each strain in that condition (left, untreated plain M9Plus media; right, TGC treated with 0.06 µg ml^−1^ TGC). The diagonal *y*=*x* line represents equal gene abundance in both strains, and *R*^2^ linear regression fits to this line are indicated. Points are coloured based on within-strain significant differential expression upon TGC treatment. See Animation S1 for an animation of these transcriptional responses and Fig. S4 for accessory gene transcriptomic responses and a version of this plot with colours separated.

Given the stress imposed on growth by TGC, even at subinhibitory levels, we predicted that transcriptional responses to this drug would be largely conserved between strains, likely consisting of stress-response genes, drug resistance mechanisms or ribosomal genes specific to the mechanism of action of TGC [65,68]. We compared transcript levels following growth in subinhibitory TGC (0.06 µg ml^−1^) to growth in minimal media lacking antibiotics. The defined minimal media used here and throughout were M9 salts buffered and supplemented with glucose, amino acids and other elements as described in the methods [42]. As expected, TGC stress caused large transcriptional changes biassed toward downregulation in both strains, but there was surprisingly minimal overlap among differentially expressed genes between strains (Fig. 1a, b). Only 144 genes, representing 24% of the total downregulated genes, and 53 genes, representing 18% of upregulated genes, were differentially expressed in the same direction in both strains. One might intuit that the limited shared response may have resulted from transcriptional differences among strain-specific genes, but this was not the case. Rather, most genes that were differentially expressed and unique to each strain were within the shared genome (Fig. 1a, b; Data S1). Many of the genes demonstrating the greatest magnitude of change were in the shared genome but were uniquely differentially expressed in each strain (Figs S2 and S3), further indicating that the response to TGC stress is historically contingent.

To test whether baseline transcriptional differences explained varied strain responses to TGC, we compared transcript abundance of conserved genes in the absence of drug (Fig. 1c, left). This comparison revealed differences in baseline transcriptional state that, upon TGC pressure, caused the transcriptional landscape of the two strains to converge upon a common pattern of gene usage (Fig. 1c, right). Genes with higher basal transcription in each strain were preferentially downregulated within that strain in response to TGC treatment (Figs 1c and S4), producing a more conserved transcriptional state under TGC pressure (Animation S1). These historical differences in basal gene expression explain the strain-dependent transcriptional responses to TGC pressure.

### Experimental evolution for tigecycline resistance results in strain-dependent trade-offs between fitness and resistance

**Fig. 2.**
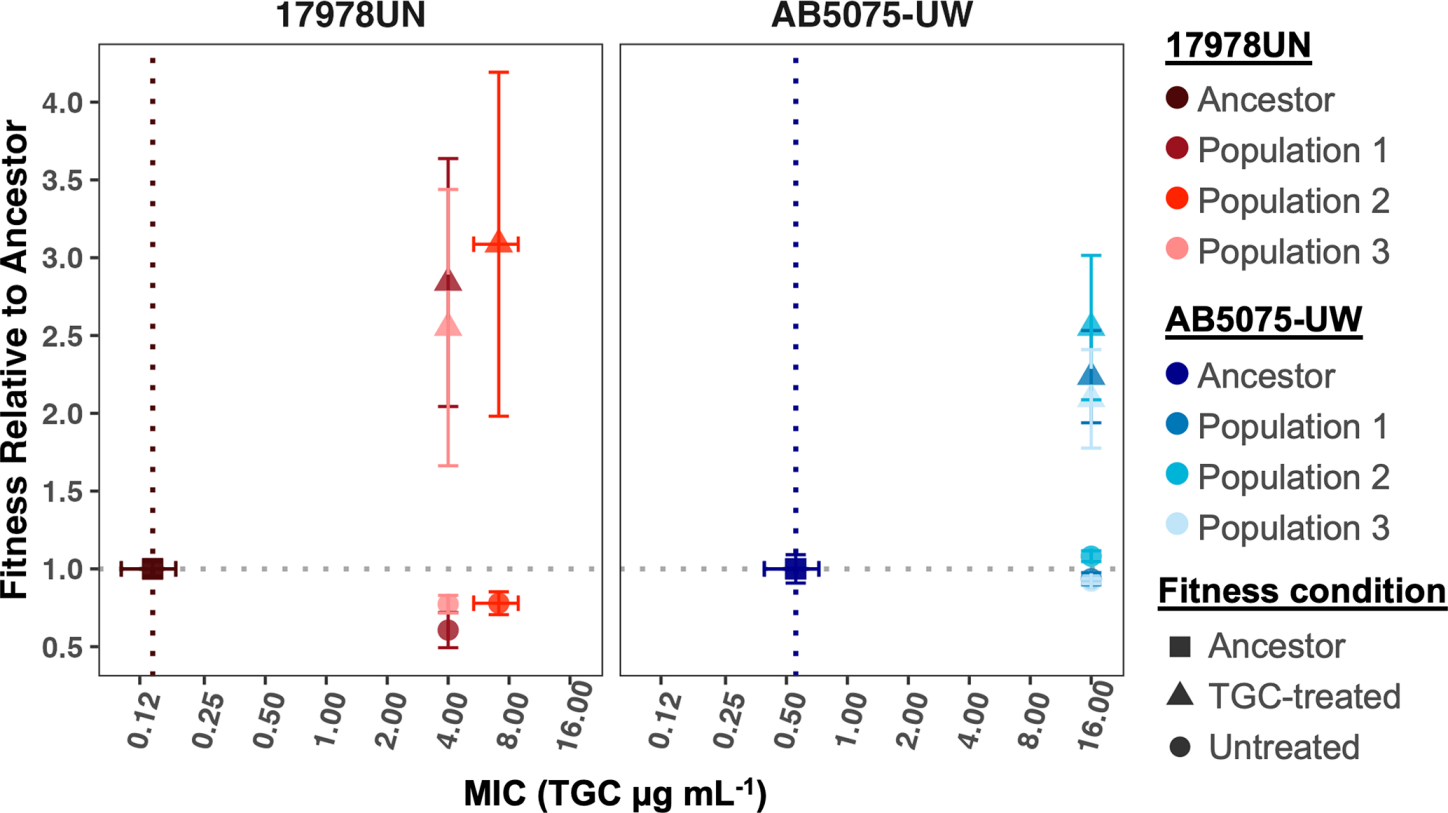
Strain-specific patterns in fitness–resistance trade-offs. MIC to TGC was measured for ancestors and day-12 TGC-evolved populations. Fitness relative to ancestral growth in each condition was calculated from growth curves as described in the methods. Fitness of 1.00 (grey horizontal dotted line) indicates the population grew equally well to its respective ancestor in that environment. Point shapes represent the conditions under which fitness was measured (untreated M9Plus, TGC treatment at 0.06 µg ml^−1^ or the ancestral growth normalized at 1). Points represent the mean across replicates, and error bars show the standard deviation of the mean.

Given the genomic signatures of history seen in the transcriptional response to TGC pressure, we hypothesized that evolutionary adaptation to this antibiotic would proceed along different paths in each strain. We propagated three replicate populations of both strains for 12 days in increasing concentrations of TGC (methods and Fig. S5). Adaptation to the media was likely minimal as control lineages of each strain evolved in the absence of TGC exhibited no mutations that reached above 20% frequency throughout the experiment. Therefore, this study focused solely on the lineages that evolved in the presence of TGC treatment. All populations evolved excess resistance beyond the drug concentration in the media, which was maintained throughout the experiment, such that all populations became nearly five times more resistant to TGC than their respective ancestors (Fig. 2).

Antibiotic resistance is often associated with a fitness cost, and these costs can depend on the strain background [17,69,71]. We expected to find deficits in absolute fitness measured via growth curves of TGC-evolved populations when grown in the absence of drug, whereas we expected higher fitness of TGC-evolved populations than their respective ancestors when grown in subinhibitory TGC. As expected, both sets of evolved populations exhibited substantial fitness increases in subinhibitory TGC. However, evolved fitness responses in the absence of drugs differed. Populations derived from the laboratory reference strain, 17978UN, incurred a 25–50 % reduction in fitness, whereas no measurable fitness cost was observed for populations derived from the more recently isolated clinical strain, AB5075-UW (Fig. 2). These results show that evolutionary trade-offs between fitness and resistance to TGC differ by founding strain and suggest that contemporary MDR strains may adapt to new drugs with limited collateral fitness costs.

### Varying population dynamics within conserved mechanisms of TGC resistance

**Fig. 3.**
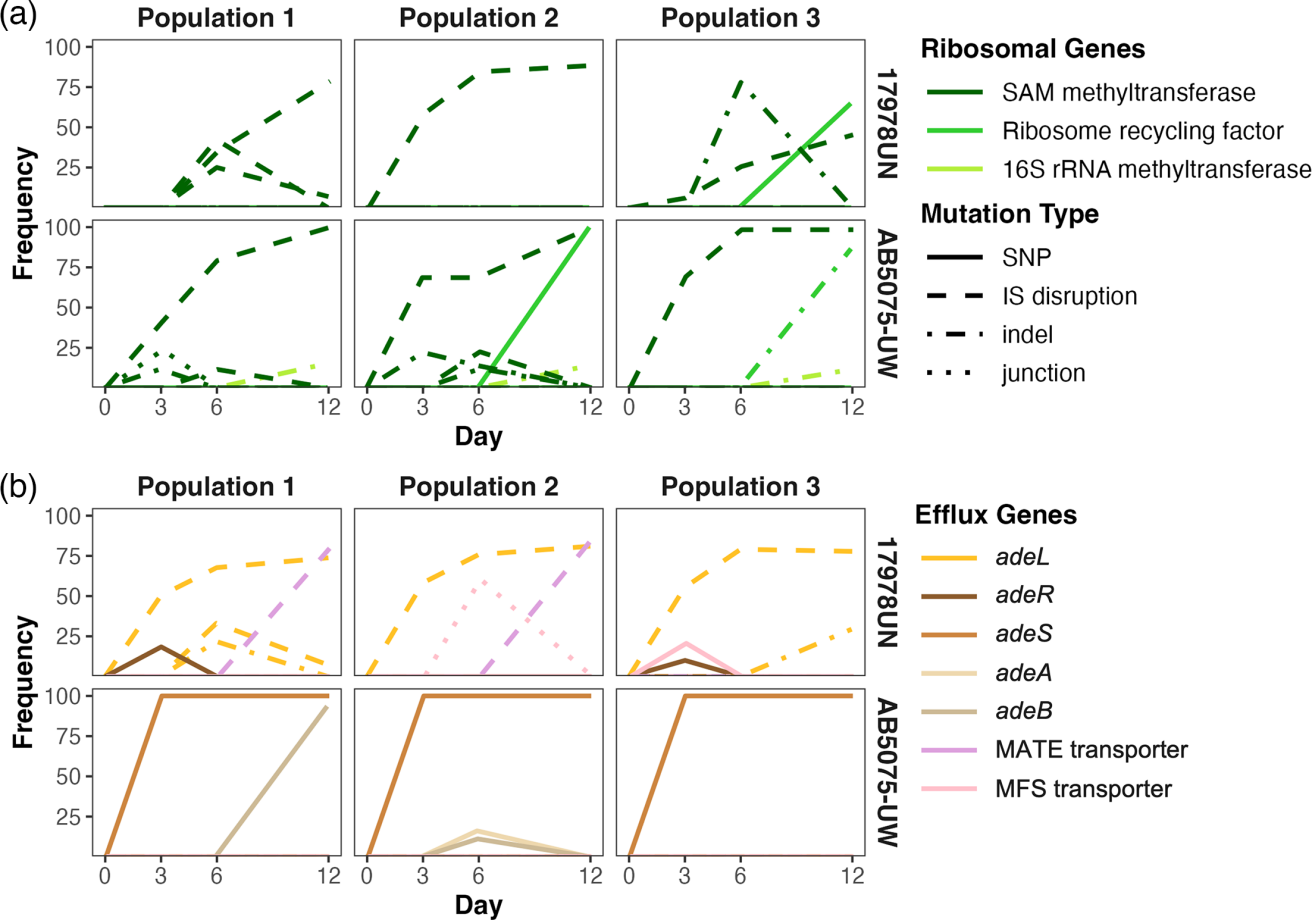
Whole-population, whole-genome sequencing reveals similar resistance mechanisms but different population dynamics between strains. Evolved mutation frequencies in TGC evolved populations over time, with line colours corresponding to affected gene and line patterns indicating mutation type. Abbreviations: IS, insertion sequence; indel, insertion or deletion; junction, structural variant indicated by a new junction call. Multiple lines of the same colour indicate that more than one unique mutation arose in that gene. (**a**) Mutations in genes associated with the ribosome. (**b**) Mutations in genes associated with efflux pumps.

The divergent relationships between fitness and resistance indicated that the genetic pathways to TGC resistance may differ between the two strains. We used longitudinal whole-population, whole-genome sequencing to investigate the population-genetic dynamics of adaptation. Following prior evolution experiments in antibiotics [42,51], we predicted that antibiotic pressure in well-mixed populations would be sufficiently strong that mutations providing resistance should rapidly sweep within the population. Indeed, mutations in genes associated with drug resistance rapidly reached fixation in all populations of AB5075-UW (Fig. 3, Data S2). Surprisingly, no individual mutations reached fixation in populations of 17978UN. This indicates that multiple adaptive lineages evolved within populations of 17978UN. A closer inspection of the mutations in each lineage suggests that in total, they represent a soft sweep of resistance-associated mutations. Soft sweeps occur when multiple independent sub-lineages acquire mutations in the same genes; individual mutations never reach fixation, but the sum of all co-existing analogous mutations in a gene or pathway approaches fixation within the population [72]. Allelic dynamics provide insight into the rate of adaptation in both of the strains as well as the relative fitness benefit of resistance mutations in a complex population. Resistance-associated mutations reached fixation by day 3 in lineages of AB5075-UW, suggesting exceptionally rapid adaptation and large fitness benefits in the presence of TGC. Adaptation in lineages of 17978UN was slightly slower, perhaps due to interference and competition of multiple alleles, with most resistance-causing mutations nearing their highest frequency by day 6.

The evolved mutations pointed to two primary mechanisms of resistance to TGC: modification of the enzymatic target of the drug, the ribosome, and decreasing the intracellular concentration of the antibiotic, primarily through efflux pumps. All populations of both strains featured high-frequency mutations affecting the same drug target modification pathway, with high gene-level parallelism. This gene encodes an *S*-adenosyl-methionine-dependent methyltransferase (SAM-MT) (Fig. 3a) that has been associated with creating methylation patterns required for TGC acting on the ribosome [73]. Interestingly, all SAM-MT mutations that reached high frequency under TGC stress were gene disruptions caused by mobilized insertion sequences that are predicted to eliminate function (Data S2). Although each population acquired mutations in the same SAM-MT, the specific insertion sequence causing the loss of function as well as the site of insertion varied among lineages, with more variability within populations of AB5075-UW. In addition to the SAM-MT, other genes related to the target of TGC acquired mutations, such as ribosome recycling factors (Fig. 3a, Data S2) that likely aid in rescuing stalled ribosomes [63].

All populations also evolved mutations in regulators of efflux pumps, but the specific resistance-nodulation-division (RND) pump that was affected differed between strains, as described below. Populations of 17978UN also acquired mutations affecting other systems predicted to reduce intracellular concentrations of TGC. For instance, two 17978UN populations acquired mutations in major facilitator superfamily transporters that rose to high frequency (Fig. 3b, Data S2) [74]. In summary, the identities, timing and frequencies of evolved mutations differed between strains, whereas replicate populations founded by the same strain exhibited high gene-level parallelism.

### Strain-dependent efflux pump preferences

**Fig. 4.**
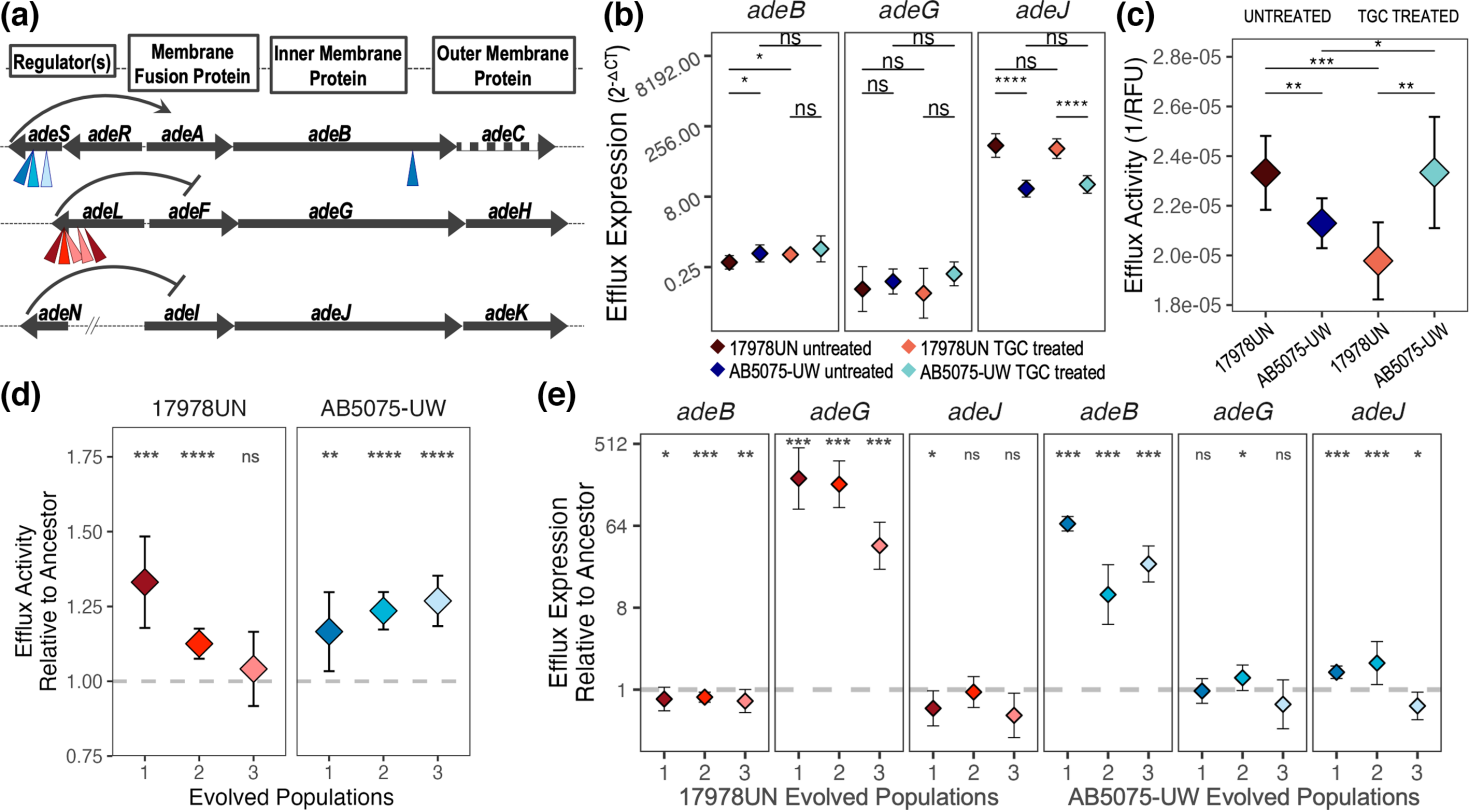
Genetic distinctions of evolved efflux expression and activity. (**a**) Representation of the RND efflux pump operons/regulators. All RND genes are highly conserved between the two strains (Table S2), except that 17978UN does not encode *adeC*. Arrow size is scaled to the length of the gene. Inverted triangles depict mutations present on day 12, coloured by the population in which they are found. Ancestral efflux activity (**b**) and expression (**c**) in treated (0.06 µg ml^−1^ TGC) or untreated states. (**d**) The efflux activity of the evolved populations when grown untreated relative to the respective ancestor. (**e**) Efflux expression in evolved populations normalized by the respective untreated ancestor. Points represent the mean and error bars depict standard deviation. Statistically significant differences were determined with t-tests in B and C, with the comparisons depicted by lines and with Wilcoxon tests compared to an ancestral value set at one in (d) and (e). Significance criteria: ns for *P*>0.05, * for *P*≤0.05, ** for *P*≤0.01, *** for *P*≤0.001, and **** for *P*≤0.0001.

All populations adapted to TGC through mutations in drug efflux systems (Fig. 3b), yet different strains evolved with different genetic signatures. *A. baumannii* encodes three RND efflux pumps known to be associated with drug resistance adaptation [75,76]. Two-component system *adeRS* regulates efflux operon *adeABC,* while the other two pumps, *adeFGH* and *adeIJK*, are regulated by negative regulators *adeL* and *adeN*, respectively (Fig. 4a) [58]. Both strains encode all three pumps with a high level (97–100 %) of amino acid conservation (Table S2), with the only exception that 17978UN does not encode the outer membrane protein, AdeC, but AdeK can supplement function [75]. Within each strain, we observed high gene-level parallelism in adaptation through mutations to RND efflux systems. Populations of AB5075-UW acquired nonsynonymous mutations throughout the *adeRSABC* efflux system, altering *adeA*, *adeB* and *adeS*. One of the two predominant SNPs in *adeS* (R152K and G160S) was fixed in all AB5075-UW populations, and an additional SNP in *adeB* (E939Q) was nearly fixed in one population (Data S2). In contrast, each evolved 17978UN population acquired mutations in *adeL* caused by an insertion sequence (IS) disruption in this gene that rose to high frequency (Fig. 3b, Data S2). This disruption exhibited further parallelism, in a site-specific manner, in which the mutation was identical within all three TGC-evolved populations, but never reached 100% frequency. As an example of the soft sweep dynamics described previously, in evolved population 3, we observe an additional mutation in *adeL*, and the two mutations in this gene, cumulatively, reach fixation. Interestingly, a nonsynonymous SNP in *adeR* also arose in 17978UN populations 1 and 3 on day three, but it was quickly outcompeted by *adeL* mutations (Fig. 3b), further emphasizing the strain-dependence of efflux mutations.

There are many ways in which historical contingencies could cause such strain-specific preferences of efflux targets. One possibility is that the sequences of these target genes had already differentiated, but all sites that acquired adaptive mutations were initially identical in the ancestral genomes (Table S3). Another possibility is that the ancestral strains had previously evolved differential regulation of these pumps by other mechanisms. To test this notion, we measured expression of efflux genes in the presence and absence of subinhibitory TGC but found comparable levels of expression of *adeABC* and *adeFGH* (Fig. 4b), discounting this explanation. We found that 17978UN exhibits relatively greater expression of the *adeIJK* pump in both growth conditions, but this pump was not the target of selection in either strain. In the absence of TGC, 17978UN had increased efflux activity measured via the ethidium bromide efflux assay compared to AB5075-UW (Fig. 4c), possibly due to the differences in AdeIJK expression. Interestingly, drug pressure induced significantly different states of efflux activity, increasing efflux activity in AB5075-UW but decreasing it in 17978UN (Fig. 4c). As expected, there were significant differences in efflux activity and expression in the evolved populations, confirming that the mutations in efflux regulators produced a meaningful phenotype. All evolved populations, except 17978UN evolved population three which still trended higher, evolved significantly increased efflux activity than their respective ancestors (Fig. 4d). Lastly, we show that the mutated regulators specifically increase expression of their associated pump (Fig. 4e). In summary, neither sequence divergence nor functional differences in the primary targets of selection explained the alternative evolutionary pathways to increased drug efflux. These results strongly suggest that more complex interactions between efflux systems and the genome favour different routes to drug adaptation.

### Diminished transcriptional response following TGC adaptation

**Fig. 5.**
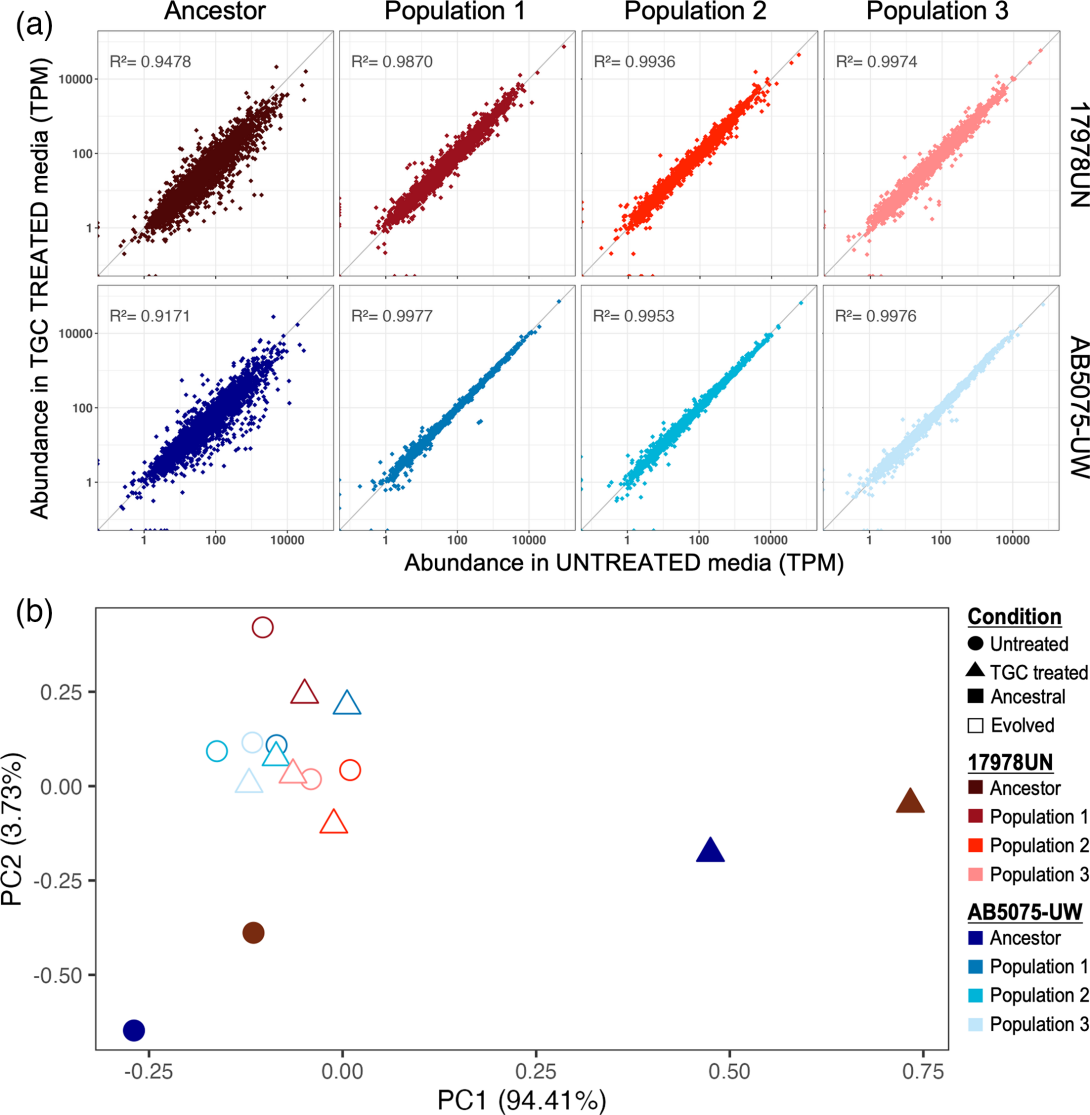
Transcriptional response evolves to minimize reaction to TGC pressure. (**a**) Conserved transcript abundance regardless of TGC treatment in all evolved populations. Each point represents the abundance of one gene in each sample. Point position depicts expression in each condition (untreated M9Plus or 0.06 µg ml^−1^ TGC). The top row shows ancestor and TGC-evolved populations for 17978UN, and the bottom row is the same for AB5075-UW. The diagonal *y*=*x* line represents equal gene abundance in both conditions, and *R*^2^ linear regression fits to this line are indicated. (**b**) Principal component analysis shows that ancestral transcriptomes are strain dependent, but TGC treatment produces a conserved shift. Evolved population transcriptional adaptation is conserved between strains and is indifferent to TGC treatment. Point colour indicates sample, shape indicates culture condition either untreated or 0.06 µg ml^−1^ TGC treated and fill indicates either ancestral or evolved populations.

The ancestral strains exhibited divergent transcriptomic changes in response to TGC stress (Fig. 1), but both adapted to TGC via broadly similar mechanisms. To evaluate the transcriptional signature of evolved drug tolerance, we conducted RNA sequencing of evolved populations grown with or without TGC treatment and found remarkably few differences (Fig. 5a). Compared to the ancestral response, where many genes changed in abundance upon TGC treatment, all evolved populations exhibited nearly identical transcriptomes in the two conditions. This trend is more conspicuous in populations of AB5075-UW, where no genes are significantly different (*P*-value<0.05 and absolute value log fold change >1) between the two conditions, while a few genes remain significantly differently expressed upon TGC stress in populations of 17978UN (Fig. S6, Data S3).

While the muted transcriptional response to the drug is observed in all evolved populations, it does not tell us if evolved transcriptional responses are conserved between strains. Principal component analysis of transcript abundance confirmed that TGC treatment shifts the initially diverging ancestral transcriptomes in the same direction while still preserving strain differences (Fig. 5b). Transcriptomes of evolved populations, either treated or untreated, strongly grouped together. This cluster of evolved population groups closely with the untreated ancestors, further supporting a lack of evolved transcriptional response to TGC stress (Figs 5b and S6). Taken together, these convergent evolved transcriptomes show that genomic drug adaptations alleviate the need to transcriptionally respond to TGC pressure and result in reduced historical signatures on the transcriptome compared to the diverging ancestral transcriptomes.

## Discussion

AMR is a problem of evolution that is most often observed in retrospect, when treatment fails. We still have much to learn about how AMR evolves, including the diversity of genetic pathways to resistance [22], the extent to which these pathways depend on the environment [42], including the host immune status [77], and whether these pathways differ among historically divergent strains [18,20]. In this study, we focused on this last factor by evaluating how reference strains, isolated nearly 60 years apart, differ in their adaptation to a clinically significant drug. Although both strains of *A. baumannii* adapted to grow in media containing TGC via common pathways, the specific genes and mutations differed between the founding strains. On the other hand, replicate populations derived from the same strain evolved in parallel, indicating a level of genetic predictability within but not between strains.

Perhaps the most striking difference between strains was the fitness consequence of TGC adaptation: lineages derived from the modern clinical reference strain experienced no measurable fitness costs, whereas those derived from the more archaic laboratory reference strain, 17978UN, incurred deficits of 25% or more (Fig. 2). Fitness effects are often related to the stability or persistence of resistance. Costly resistance has long been proposed as a beneficial trait for humanity that can preserve drug efficacy in the face of selection [3,78, 79]. Here, we find an alarming lack of a fitness cost of evolved resistance in lineages derived from the contemporary clinical isolate, AB5075-UW. The evolutionary history of AB5075-UW includes frequent antibiotic exposure, as well as likely selection in the absence of antibiotic pressures, which could have selected for compensatory adaptations that alleviate the costs of TGC resistance [80]. While sparsely studied in clinical MDR isolates of *A. baumannii*, other studies have found that positive epistasis between pre-existing resistance alleles can ameliorate further costs upon acquisition of additional resistance [81,83]. Discerning the epistatic mechanism reducing the fitness cost in TGC-resistant AB5075-UW populations would require further investigation. The lack of a fitness–resistance trade-off suggests that passive loss of resistance once drug pressure is alleviated may be rare, increasing the need for alternative treatment options.

Differences in the genetic causes of resistance between strains offer evidence that historical contingency influences routes to AMR. Many bacterial genomes, including *A. baumannii,* encode multiple RND efflux pumps, and to explain this apparent redundancy, a hierarchical model of sequential activation has been proposed [58,74, 84]. We find that the first, dominant mutations affecting drug efflux in each strain affected regulators of different pumps, with SNPs in *adeS* sweeping to fixation in AB5075-UW populations and gene disruptions caused by IS in *adeL* reaching high frequencies in 17978UN populations (Fig. 3). This suggests that the hierarchy of these pumps differs between strains in the face of TGC pressure and raises the possibility that variation in their regulatory network or gene content evolved in the past. Perhaps the diminished priority of the AdeRSABC pump in 17978UN stems from the lack of an encoded *adeC* in this strain (Fig. 4a, Data S1). These lineages would need to accumulate two mutations to overexpress one functional pump: one that upregulates AdeAB and one that upregulates AdeK to serve as the outer membrane protein [75]. The historical contingency imposed by the loss of *adeC* sometime in the history of 17978UN may be driving the strain-dependent efflux preferences for resistance adaptation.

Both the spectra of evolved mutations and their dynamics differed between strains, suggesting intrinsic biases that influence genetic diversity. Despite having the same number of encoded IS as AB5075-UW (Table S1), nearly all high-frequency mutations in populations of 17978UN were caused by IS interrupting open reading frames, whereas populations of AB5075-UW experienced a greater diversity of mutation types. IS elements are known to play an important role in *A. baumannii* mutagenesis [85]. The strain-dependent historical contingencies on insertion sequence mobilization, repertoire and insertion site could have lasting impacts on the future stability of these mutations and, therefore, the stability of resistance stemming from IS-mediated mechanisms [85,86]. Further, resistance-associated mutations were fixed in populations of AB5075-UW, but no mutations were fixed in populations of 17978UN, indicating coexisting lineages. The maintained diversity in 17978UN lineages may be due to relatively greater mutation availability causing the coincident rise of multiple adapted lineages. When multiple mutations in the same gene collectively reach fixation, this phenomenon is known as a soft sweep [87,88]. At the population level, the diversity maintained in a population undergoing selection from antibiotics is an important indicator of future paths available for additional adaptation in new environments. In this regard, populations of 17978UN may be more evolvable when challenged with a new stress [89].

It would be ideal to be able to predict evolutionary trajectories of AMR from patterns of transient response to an antibiotic. While we do not see clear signatures that may enable predictions of drug adaptation at the gene level [65], our work shows that strain background greatly influences transcriptomic response to antibiotics. The large transcriptomic differences between strains in the absence of drug, which in turn influenced their responses to TGC (Fig. 1), were remarkable and could be used to map functional contingencies between strains. Many more transcriptomes of additional strains would be needed to evaluate the power of transcriptomes as markers of evolutionary history and to anticipate drug responses. Nonetheless, our studies suggest the existence of a conserved transcriptional state under TGC stress that different unexposed cell states migrate toward. The conserved transcriptomes of evolved populations harbouring several new AMR mutations further support a homogeneous state associated with drug adaptation (Fig. 5). We noted that the evolved transcriptomes more closely represent the untreated ancestral state, suggesting that the evolved populations are, essentially, not feeling the pressure of the antibiotic, likely due to the adaptations in drug efflux decreasing the intracellular concentrations of TGC (Fig. 4). It would be valuable to test if the collapse of transcriptional heterogeneity upon transient antibiotic pressure, as well as prolonged adaptation, is a general pattern or is contingent on the class or concentration of antibiotic applied. A lack of transcriptional response to antibiotics in clinically adapted samples could be used as another biomarker upon which to predict the extent of genetic resistance adaptation.

In a world shifting toward personalized medicine, we should also shift the treatment of infections to be tailored to the specific strain or sequence type causing the infection. This work shows that initial responses, as well as evolutionary trajectories, to antibiotic stress can be highly strain-dependent, likely influencing treatment efficacy and differential persistence of MDR organisms in hospital systems. If we are better able to predict the impact of historical contingencies in the transcriptional response and fitness trade-offs associated with AMR evolution, we will be one step closer to personalized treatment of MDR infections.

## Supplementary material

10.1099/mic.0.001570 Uncited Supplementary Material 1.

## Abbreviations

AMR: antimicrobial resistance
AUC: area under the curve
IS: insertion sequence
MDR: multidrug-resistant
RND: resistance-nodulation-division
SAM-MT: *S*-adenosyl-methionine-dependent methyltransferase
TGC: tigecycline
