# Supplementary material for: History shapes regulatory and evolutionary responses to tigecycline in two reference strains of Acinetobacter baumannii
Source: Microbiology (Reading). 2025 Jun 9;171(6):001570. doi: 10.1099/mic.0.001570 (PMC12149411; doi:10.1099/mic.0.001570)
Supplement: Uncited Supplementary Material 1. [file mic-171-01570-s001.pdf]

Supplemental Materials for "History shapes regulatory and evolutionary responses to tigecycline in two reference strains of *Acinetobacter baumannii,*" by Alecia B. Rokes, Alfonso Santos-Lopez, and Vaughn S Cooper

1. **Supplemental Animation**
2. **Supplemental Figures S1-S6**
3. **Supplemental Tables S1-S4**
4. **Supplemental Data S1-S3**
5. **Supplemental Methods**
6. **Supplemental References**

SUPPLEMENTAL ANIMATION

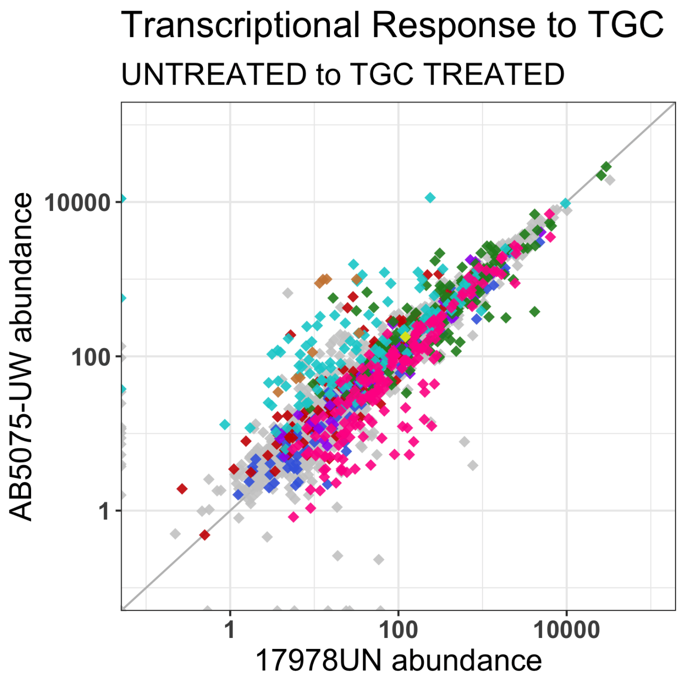

Animation S1: Tigecycline treatment induces a shift toward a more conserved transcriptional response. Animation of main text figure 1, showing movement of genes from the untreated state to the TGC treated state. See figure 1 for color key and legend. Motion version of Animation S1 can be found at https://github.com/vscooper/tigecycline

SUPPLEMENTAL FIGURES

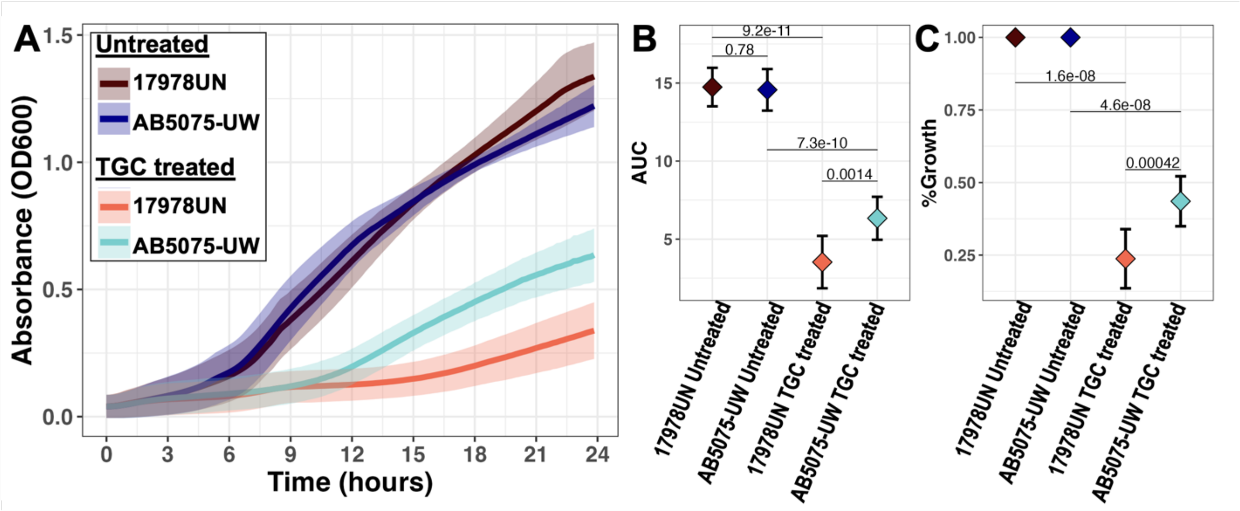

Fig. S1: Ancestral growth in treatment conditions. Growth was measured in minimal M9plus media (Untreated) or M9plus with addition of 0.06 µg/mL TGC (TGC treated) with three biological replicates (batches), each with three technical replicates. (*A*) Growth curves for ancestors grown in different conditions. Solid line represents mean, and ribbons depict standard deviation. (*B*) Area under the curve (AUC) quantifications of curves in A (used to normalize evolved population growth in Fig. 2). (*C*) Treatment effect on ancestral growth. Y-axis shows the percent growth defect caused by TGC treatment on each strain, normalized by within-batch growth in plain media. For B and C, mean and standard deviation depicted by point and error bars, p-values calculated from pairwise t-test.

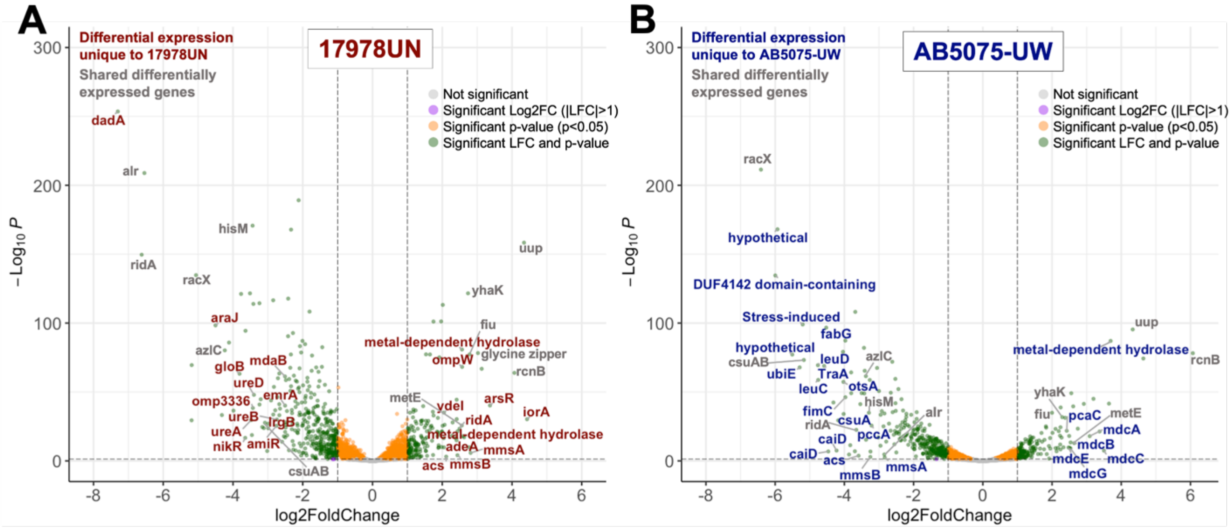

Fig. S2: Transcriptional response to TGC is strain dependent. Volcano plots of differential expression in 17978UN (*A*) and AB5075-UW (*B*). Points are colored based on significance status and fold change; Labels are colored based on if the gene is significantly differentially expressed in only 17978UN (red), in only AB5075-UW (blue), or significant in both strains (gray). Labels were manually curated to label the strongest hits or hits that showcase differences between the strains. RNAseq was done in triplicate for each strain x treatment. Genes were included as significant in the counts/plots if they had an absolute value of log 2-fold change > 1 and a p-value ≤ 0.05.

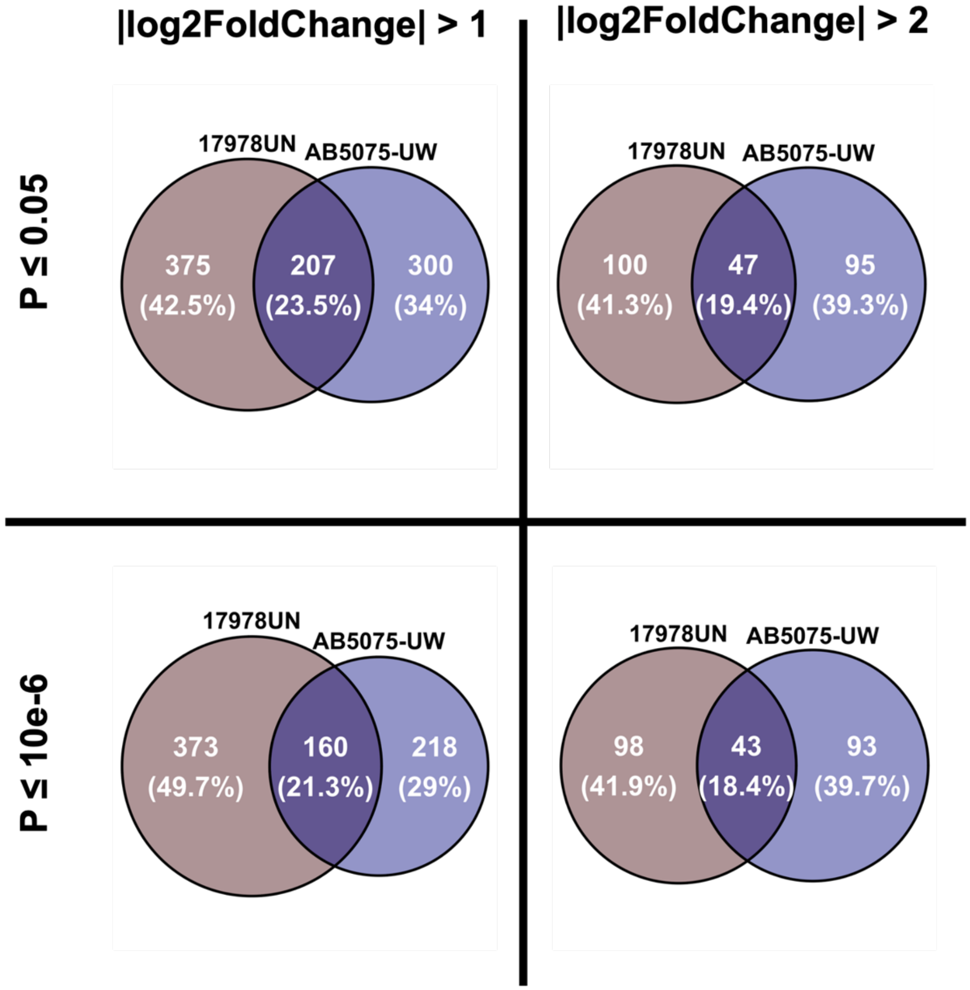

Fig. S3: Significance cut-off criteria does not affect amount of differential expression overlap between strains. All differentially expressed genes (both upregulated and downregulated) in response to TGC treatment were summed. Varying cut-offs for calling significance in p-value and in log 2-fold change were tested to determine if the patterns of differential expression overlap between strains would differ. The overlap in differentially expressed genes between strains slightly decreases as increasingly strict significance criteria are applied.

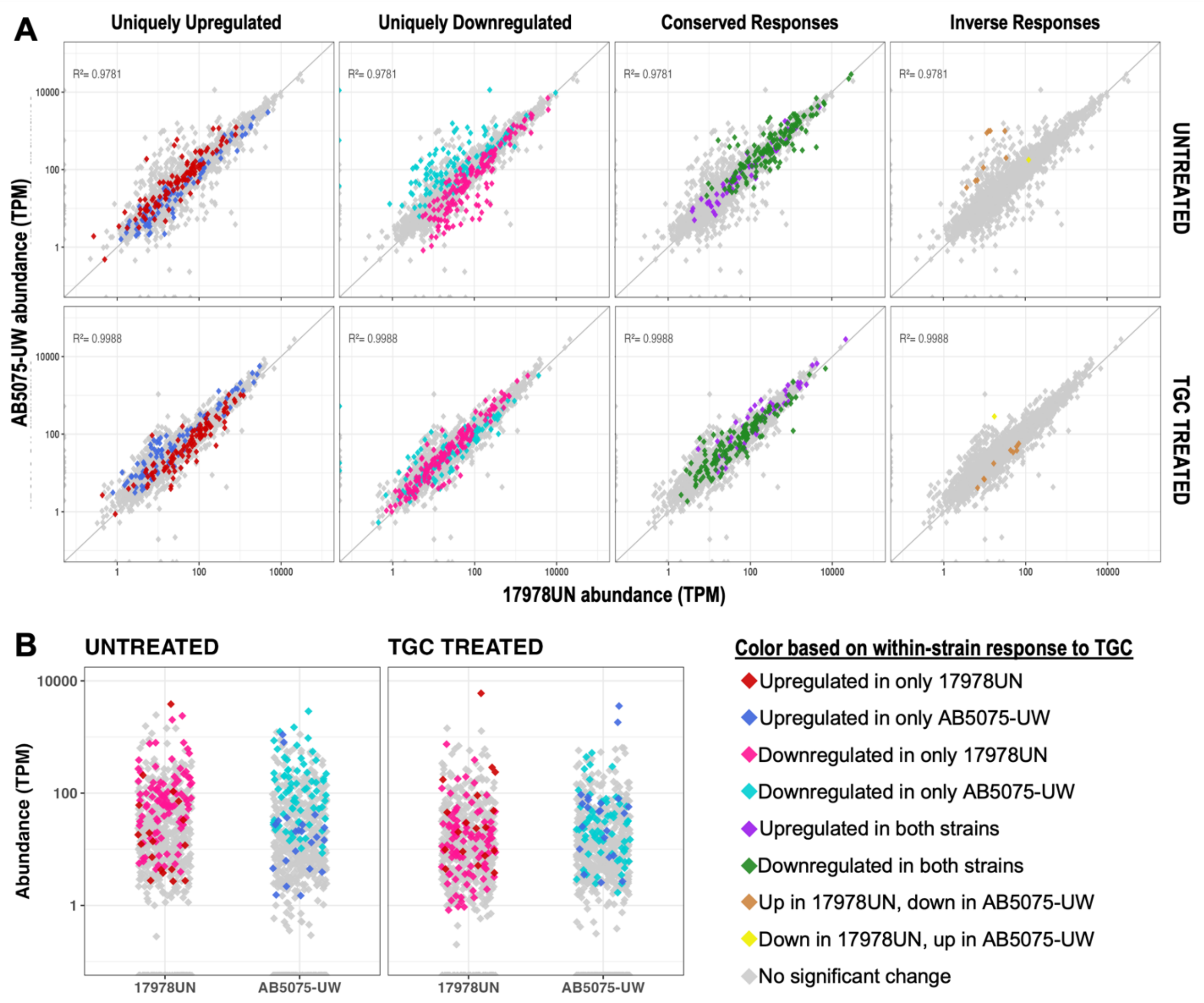

Fig. S4: Strain-dependent transcriptional response to TGC results in a shift toward a more conserved transcriptional state. (*A*) Specific directional gene responses with genes colored based on how that gene responds within-strain upon TGC pressure. The top panels show gene abundances in untreated media and bottom panels show gene abundance in TGC treatment (0.06 µg/mL). Only genes falling under the bin of directional response noted above the plots are colored. All other genes, even those with significant TGC responses, are gray in these plots. (*B*) Abundance plots of the accessory genes, using the same colors as in A and in main text figure 1. See figure 1 legend for more details.

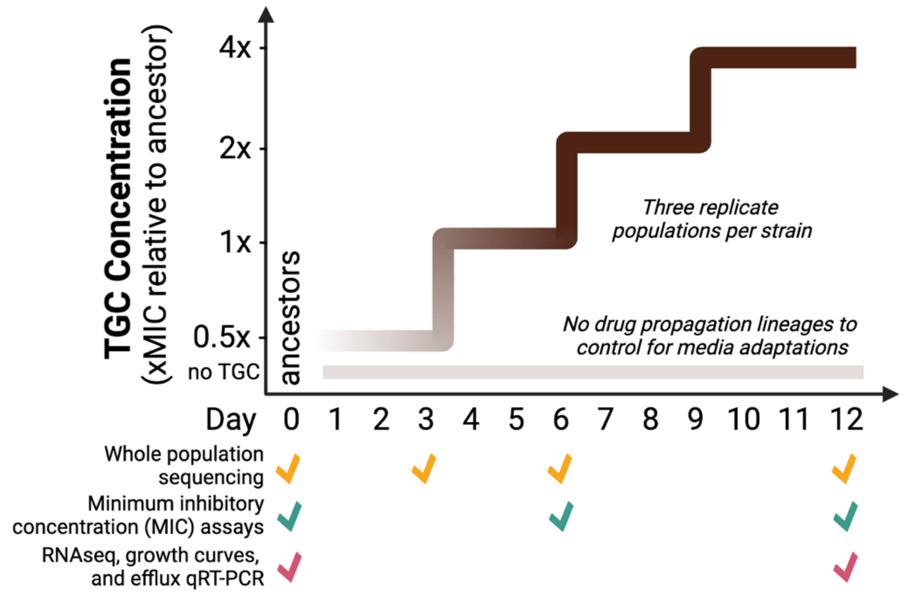

Fig. S5: Experimental design and post-experiment assay timings. Schematic for the design of the experimental evolution. For in-depth design, see the methods section. Bottom panels highlight when various phenotype/genotype assays were performed. Dark steps indicate TGC concentration doubling every three days of the experiment. Faint bar on bottom represents lineages propagated in M9plus media lacking antibiotic to assess media adaptations.

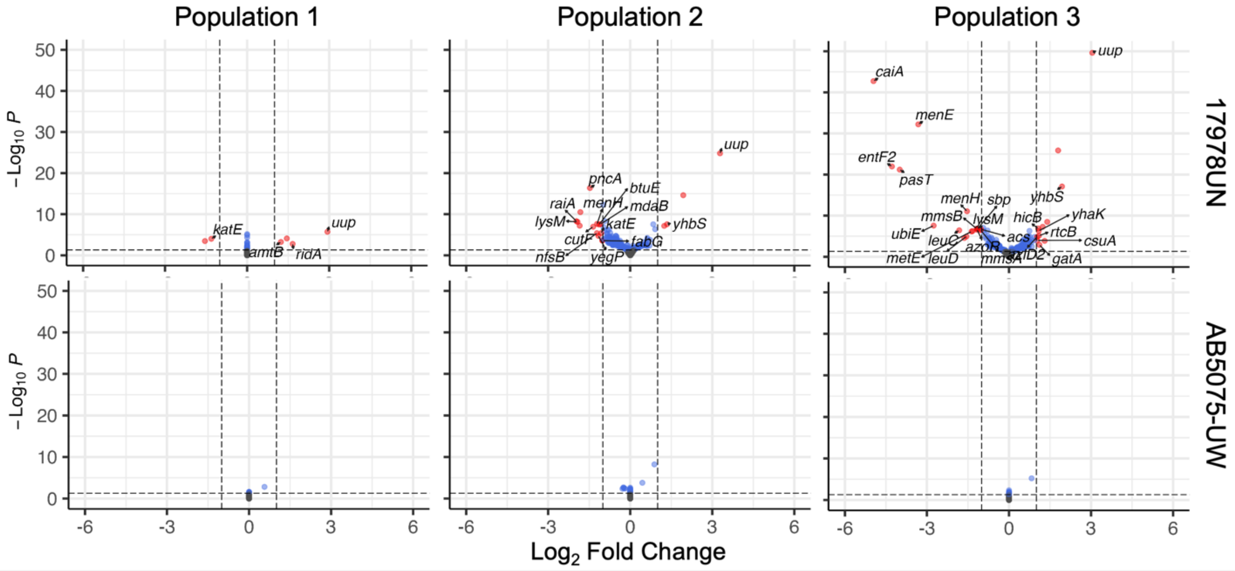

Fig. S6: Transcriptional response to TGC in evolved populations is minimal, especially in lineages of AB5075-UW. A handful of genes significantly respond to TGC treatment (0.06 µg/mL), compared to growth in untreated media, in evolved populations of 17978UN (*top*), but no genes significantly respond to TGC in any evolved population of AB5075-UW (*bottom*). All samples are from day 12 of the TGC evolution experiment. Data is from averages of three biological replicates for all sample x treatment combinations except for removal of two replicates due to low sequencing coverage or outlier status (17978UN population 1 untreated replicate A and AB5075-UW population 2 untreated replicate C). Differential expression significance cutoffs: |LFC|>1 and p-value ≤ 0.05.

SUPPLEMENTAL TABLES

Tables S1-S4 can also be found at https://github.com/vscooper/tigecycline

Table S1: Genomic comparisons between strains. Summary of strain similarities and differences in various genomic categorizations, including gene count in clusters of orthologous group (COG) categories, total genes assigned COG categories, AMR finder found resistance elements, and annotated insertion sequences. A proportional z-test was performed for all appropriate comparisons with the relevant gene count as the proportion denominator.

		AB5075-UW	17978UN	p-value (proportional z-test)
COG Categories	Cell cycle control, cell division, chromosome partitioning	34	37	1
	Cell motility	40	44	1
	Cell wall, membrane and envelope biogenesis	170	188	0.957
	Cytoskeleton	2	2	1
	Defense mechanisms	61	75	0.547
	Extracellular structures	36	39	1
	Intracellular trafficking, secretion, and vesicular transport	58	60	0.832
	Posttranslational modification, protein turnover, chaperones	115	128	0.94
	Signal transduction mechanisms	108	122	0.855
	Replication, recombination and repair	87	104	0.583
	RNA processing and modification	1	2	1
	Transcription	251	287	0.619
	Translation, ribosomal structure and biogenesis	217	224	0.561
	Amino acid transport and metabolism	276	278	0.33
	Carbohydrate transport and metabolism	126	126	0.508
	Coenzyme transport and metabolism	155	170	1
	Energy production and conversion	164	170	0.654
	Inorganic ion transport and metabolism	165	191	0.619
	Lipid transport and metabolism	197	219	0.902
	Nucleotide transport and metabolism	84	84	0.61
	Secondary metabolites biosynthesis, transport and catabolism	55	68	0.553
	Function unknown	113	130	0.736
	General function prediction only	210	232	0.951
	Total number of genes with COG assignments	2725	2980	**1.55E-12**
	AMRfinder Plus found elements	19	6	**0.017**
	Annotated IS elements	14	14	1

Table S2: Resistance profiles for the laboratory reference strain, 17978UN, and the multi-drug resistant clinical reference strain, AB5075-UW. Susceptibility/resistance state was measured in duplicate for each strain to a variety of Gram-negative acting antibiotics via Sensititre plates #GN3F (ThermoFisher). Sensitive (S), intermediate (I), and Resistant (R) classifications were assigned using CLSI standards for Gram-negative bacteria. *Tigecycline was chosen for further use in this study and broth-dilution MICs were determined to differ 1-fold between the strains.

Antibiotic	17978UN	AB5075-UW
Amikacin	≤ 8 µg/mL	32 µg/mL
	S	I
Ampicillin	≤ 4 µg/mL	> 32 µg/mL
	S	R
Ampicillin/sulbactam	≤ 4/2 µg/mL	16/8 µg/mL
	S	I
Aztreonam	16 µg/mL	> 32 µg/mL
	I	R
Cefazolin	> 32 µg/mL	> 32 µg/mL
	R	R
Cefepime	≤ 4 µg/mL	> 32 µg/mL
	S	R
Cephalothin	> 16 µg/mL	> 16 µg/mL
	R	R
Meropenem	≤ 1 µg/mL	> 8 µg/mL
	S	R
Ertapenem	≤ 2 µg/mL	> 16 µg/mL
	S	R
Cefuroxime	16 µg/mL	> 32 µg/mL
	R	R
Gentamicin	≤ 2 µg/mL	≤ 2 µg/mL
	S	S
Ciprofloxacin	≤ 0.5 µg/mL	> 4 µg/mL
	S	R
Piperacillin / tazobactam	≤ 16/4 µg/mL	> 128/4 µg/mL
	S	R
Cefoxitin	32 µg/mL	> 32 µg/mL
	R	R
Trimethoprim / sulfamethoxazole	> 4/76 µg/mL	≤ 0.5/9.5 µg/mL
	R	S
Cefpodoxime	4 µg/mL	> 16 µg/mL
	I	R
Ceftazidime	2 µg/mL	4 µg/mL
	S	S
Tobramycin	≤ 4 µg/mL	≤ 4 µg/mL
	S	S
**Tigecycline***	**≤ 1 µg/mL**	**≤ 1 µg/mL**
	**S**	**S**
Ticarcillin / clavulanic acid	≤ 16/2 µg/mL	> 64/2 µg/mL
	S	R
Ceftriaxone	8 µg/mL	16 µg/mL
	I	I
Tetracycline	≤ 0.5 µg/mL	≤ 0.5 µg/mL
	S	S

Table S3: Conservation of efflux proteins between 17978UN and AB5075-UW. Pairwise identity of amino acid sequences. * *adeC* is only encoded by AB5075-UW, but it is suggested that *adeK* can assume the function of *adeC* in 17978UN (Leus et. al., 2018). Importantly, all sites that will be mutated upon TGC adaptation are initially identical between the strains.

Protein	Length	Amino Acid Pairwise Identity (%)
AdeS	362	97.2
AdeR	248	100
AdeA	397	98.7
AdeB	1037	99.5
AdeC	466	*
AdeN	218	100
AdeI	417	100
AdeJ	1059	99.9
AdeK	485	100
AdeL	344	99.7
AdeF	407	99.5
AdeG	1060	99.9
AdeH	483	99.8

Table S4: List of primers used for Q-RT-PCR. Quantative, reverse-transcriptase PCR was performed on the inner membrane pump of the three RND efflux pumps as documented in the methods using primers with the characteristics listed here. The housekeeping gene *rpoB* was used as a control.

Target	Direction	Sequence (5'-3')	Melt Temp (ºC)	Product Size	Source
rpoB	forward	TCCGCACGTAAAGTAGGAAC	57.6	155	Coyne et al. Antimicrobial Agents and Chemotherapy
	reverse	ATGCCGCCTGAAAAAGTAAC	57		
adeB	forward	AAAGGTATTGGCTACGAGTGG	57.7	132	Lin et al. Frontiers in Microbiology. 2017
	reverse	TGCCCAGCTTTCATAGAGTG	57.6		
adeG	forward	CAAAGTCCAGCATTACCAGC	57.1	136	This study, geneious primer3
	reverse	CTGCCCCAGGTTATTCATCT	57		
adeJ	forward	TGACCAACACCTGTGAATGA	57	164	This study, geneious primer3
	reverse	GAAGATCAGGGTGTGGTCAT	56.9		

SUPPLEMENTAL DATA LEGENDS

There are three submissions for supplemental data, each a separate file that can be accessed through https://github.com/vscooper/tigecycline

Please contact authors for additional information about supplemental data.

Data S1: Ancestral gene annotations and RNA sequencing results.

Data S2: Filtered results of whole-population, whole-genome sequencing throughout the evolution experiment.

Data S3: RNA sequencing results of the evolved populations.

BIOSAMPLE ACCESSION NUMBERS

46406407	46406381	46388238
46406406	46406380	46388237
46406405	46406379	46388236
46406404	46406378	46388235
46406403	46406377	46388234
46406402	46406376	46388233
46406401	46406375	46388232
46406400	46406374	46388231
46406399	46406373	46388230
46406398	46406372	46388229
46406397	46406371	46388228
46406396	46406370	46388227
46406395	46406369	46388226
46406394	46406368	46388225
46406393	46406367	46388224
46406392	46406366	46388223
46406391	46406365	46388222
46406390	46406364	46388221
46406389	46406363	46388220
46406388	46406362	46388219
46406387	46406361	46388218
46406386	46406360	46388217
46406385	46388242	46388216
46406384	46388241	46388215
46406383	46388240	46368425
46406382	46388239	

SUPPLEMENTAL METHODS

Bacterial strains and growth conditions

Laboratory reference strain ATCC 17978UN (1,2) and clinical reference strain AB5075-UW (3,4) were used throughout the study and grown under the same conditions. Unless otherwise stated, bacterial cultures were grown in 5ml M9+ media, as previously described (5). Briefly, M9+ media is a salts-buffered media with glucose (11.1mM) as the primary carbon source. It contains 0.1 mM $CaCl_2$, 1 mM $MgSO_4$, 42.2 mM Na_2HPO_4, 22 mM KH2PO_4, 21.7 mM NaCl, 18.7 mM NH_4Cl and is supplemented with 20 mL/L MEM essential amino acids (Gibco 11130051), 10 mL/L MEM nonessential amino acids (Gibco 11140050), and 1 mL each of trace mineral solutions A, B, and C (Corning 25021–3 Cl). Cultures were incubated at 37°C with shaking or on a roller-drum at approximately 250rpm.

Genomic comparison of the two strains

Genomes were annotated with bakta (v1.6.1, database v4.0) (6) and input to Panaroo (v1.3) in the sensitive clean mode to obtain a gene presence/absence list that included plasmid encoded genes (Data S1) (7). We assessed pairwise nucleotide similarity of the two strains with pyANI (v0.2.12; (8). Multilocus sequence types were confirmed using mlst (v2.11; https://github.com/tseemann/mlst) (9). Elements associated with resistance, including known single nucleotide polymorphisms (SNPs), were detected with AMRfinderPlus (version 3.11.26 with database version 2023-11-15) (10). Two-proportion z-tests on two-tailed hypotheses were used to determine if gene content in clusters of orthologous group (COGs) categories was significantly different in the two strains, accounting for the difference in genome size (Table S1) (11). The annotated reference genomes used in this study can be found at https://github.com/vscooper/tigecycline.

Measuring resistance

Broad resistance profiles of the two strains were determined using Sensititre plates (ThermoFisher, GN3F), commercially available panels of 23 antibiotics relevant to Gram-negative pathogens at dilutions spanning the clinical breakpoint for resistance as set by the Clinical and Laboratory Standards Institute (CLSI) (12). Broth microdilution and inoculation of the plates was performed following the manufacturer's instructions. Sensititre plates were performed in biological triplicate for each strain.

Minimum inhibitory concentration (MIC) assays were performed to measure susceptibility levels more accurately to tigecycline (TGC, Sigma 220620-09-7). MIC assays were performed following modified CLSI methods (12). MIC results presented here are from MIC assays performed in M9plus media. Reference MIC assays were performed in the standard Mueller Hinton broth and showed little-to-no (maximum difference of 1-fold) difference to those performed in M9plus media. Biological triplicate (multiple samples

from the frozen ancestors or populations) with technical triplicates for each assay were performed. We present the concentration of antibiotic that inhibits 90% growth (IC_{90}), measured via optical density at 600nm, as the MIC for each antibiotic.

RNA extraction and purification

For transcriptome analysis of the ancestor strains, cultures were seeded from individual colonies in biological triplicate into 5mL M9plus media. Population cultures were started directly by inoculating a large sampling of freezer stock into 5mL M9plus, also in triplicate. All cultures were grown over-night at 37°C and back diluted 1:100 into 25 mL pre-warmed M9plus with or without 0.06 µg/mL TGC. In this way, each biological replicate over-night culture results in a paired set of treated and untreated TGC culture for analysis, thus limiting the effect of sampling bias for the populations. Cultures were grown until reaching mid-log phase (OD600 = 0.6 +/- 0.05), at which time cultures were pelleted, resuspended in TRIzol (Invitrogen Cat. No. 15596026) and stored at -80°C for subsequent extraction and purification.

RNA was extracted following a modified protocol (13) for TRIzol extraction with purification using the Invitrogen PureLink® RNA Mini Kit. Extraction included one bead beating step followed by TRIzol-chloroform extraction with ethanol precipitation. Purification was done using RNA columns from the Invitrogen PureLink system with final elution into nuclease free water. RNA samples were treated with DNase 1 off-column with additional DNase spike-in during incubation (NEB DNase and buffer Cat. No. M0303S) as well as treated with DNase 1 on-column (Qiagen Cat. No. 79254) during final purification steps on the Invitrogen columns. RNA concentration and quality were assessed using nanodrop (all 260/280 ratios > 2). Pure RNA was stored at -20°C for less than a week prior to library preparation and at -80°C for longer-term storage. Step-by-step RNA protocols are available upon request.

RNA sequencing and analysis

RNA integrity, fragment distribution, and RIN^e scores were assessed on Tapestation using the High Sensitivity RNA ScreenTape, sample buffer, and ladder from Agilent Technologies (Part Numbers: 5067 -5579, -5580, -5581). All RIN^e scores satisfied subsequent ribo-depeletion requirements for quality (the majority of RIN^e scores were >8). rRNA depletion was performed using the RiBO-COP rRNA Depletion Kit for Gram-Negative Bacteria (Lexogen Cat. No. 126.96) and directly following the manufacturer's protocols with an initial RNA input of approximately 200ng. RNA libraries including ancestral strain RNAs were prepared using the CORALL Total RNA-Seq Library Prep Kit (Lexogen Cat. No. 095) while the evolved populations were sequenced with the RNA-Seq V2 Library Prep Kit with UDIs (Lexogen Cat. No. 171.96). Both library preparations included the PCR add-on

step to determine ideal cycle number (11 cycles) for library amplification (Lexogen Cat. No. 020). The uniquely indexed samples for each library were pooled based on fragment size and concentration and the pool was diluted to 4nM for sequencing. Each library was denatured and loaded at a final concentration of 2pM with a 5% phiX spike-in. Sequencing was performed on the Illumina NextSeq550 with the corresponding High-Output Kit v2.5 75 cycles (Illumina 20024906). Raw reads are uploaded to the NCBI BioProject ID PRJNA1214285.

Reads were demultiplexed using bcl2fastq (v2.17.1) and trimmed with trimmomatic (v0.36, custom criteria as follows: LEADING:3 TRAILING:3 SLIDINGWINDOW:4:15 MINLEN:36). Read quality was confirmed with fastQC (v0.11.5) and kallisto (v0.48.0, flags as follows: --single -l 315 -s 26) was used for pseudoalignment with custom made index files from the concatenated bakta annotated fasta files previously described for each reference strain (14). Sequencing coverage was satisfactory (>10^7 reads/sample, resulting in >200x coverage) for all samples apart from one replicate of AB5075-UW evolved population 2 in the untreated condition, which had unusually low coverage and was removed from further analysis. We also removed one replicate of 17978UN evolved population 1 in the untreated condition. DESeq2 (v1.38.3; (15)) was used in RStudio (R v4.2.1) for differential expression analysis supplemented with EnhancedVolcano (v1.16.0) for plotting. Transcript abundance is reported as transcripts per million transcripts (TPM) which normalizes by both gene length and sequencing depth. TGC imposed differential expression analyses were performed with guidance from established tutorials (15,16). In the results function for analysis of the DESeq Data Set, we specified to turn off independent filtering and cooksCutoff to keep outliers and calls with low coverage in the analyses, then the apeglm method was applied for log fold change shrinkage (17). Unless otherwise stated, cutoffs for significance were set to a false discovery rate adjusted p-value < 0.05, with the Benjamini and Hochberg correction for multiple comparisons, and a magnitude of log_2FoldChange > 1.

<u>Experimental evolution with TGC</u>

Our experimental design was adapted from previous studies (5,18). Both strains were resurrected from the freezer via streaking on LB agar plates. A single colony of each strain was used to inoculate a 5mL overnight culture in M9+. After approximately 18 hours, the saturated culture was used to inoculate multiple independent lineages for the evolution experiment. We propagated five replicate lineages of each strain in each condition, either with or without TGC. Five lineages afforded us the confidence that should lineages die or be contaminated, we would be left with three per strain per condition to analyze, which is an accepted practice for evolution experiments (19–21). Lineages were inoculated from overnight culture (either the starting overnight, or the prior day) with a 1:100 dilution into fresh media with or without TGC. 1:100 dilutions from saturated

overnight cultures results in approximately 6.6 generations per day (22). The large population size combined with the 1% bottleneck, mutation rate, and doubling time of *A. baumannii* provides a large mutation supply, with a probability >1 that each site can see a mutation (22).

Lineages were propagated in the presence and absence of TGC, resulting in two conditions. The no drug lineages were used to control for adaptations to the media and experimental conditions. In the TGC treated condition, the TGC level in the media was tailored to the initial susceptibility level of each strain, starting at 0.5x the ancestral MIC (subinhibitory). Every three days, the concentration of TGC in the media was doubled (Fig. S5). We propagated the lineages for 12 days, with the final TGC media concentration at 4x the ancestral MIC, which crossed the clinical breakpoint for resistance in both strains. On days 0, 1, 3, 4, 6, 7, 9, 10, 12, corresponding to days before and after antibiotic level increases, the populations were stocked for later assays and sequencing (frozen in 9% DMSO at -80°C, and a cell pellet from 1mL of culture was frozen at -20°C).

Whole-population, whole-genome sequencing, and analysis

DNA was extracted from frozen cell pellets using the DNeasy blood and tissue kit for the QIAcube (Qiagen, Hilden, Germany) with a 10-minute elution into nuclease-free water. Libraries were prepared in-house as previously described (23,24) or using the plexWell^{TM} kit following manufacturer's directions (SeqWell PW096). Libraries were sequenced using an Illumina NextSeq550 sequencer with a 300 cycle mid-output kit (Illumina 20024905). Reads were demultiplexed, trimmed, and quality checked as described in the supplemental methods for the RNAseq reads. Raw reads are uploaded to NCBI BioProject ID PRJNA1214285. Breseq (v0.35.0) was used for read mapping and variant calling (25) with subsequent filtering following previously published rationale (18) with special attention to new junction calls. Filtering, consolidating for allele frequencies, and plotting were done in RStudio (R v4.2.1) with the packages ggplot2 (v3.4.2; https://CRAN.R-project.org/package=ggplot2) and tidyr (v1.3.0; https://CRAN.R-project.org/package=tidyr).

Growth curves as measure of fitness

We use bacterial growth curves to measure absolute fitness of ancestral clonal samples as well as to measure aggregate absolute fitness of evolved populations (26). Growth curves were seeded to mimic the transfers of the evolution experiment. A large sample of the frozen population (or ancestor) stock was inoculated, in biological triplicate, into M9plus media and grown for 24h. Overnight cultures were then diluted 1:100 into fresh media and OD600 measured every 10 minutes for 24h. Fitness was measured, in technical triplicate, in plain M9plus as well as in M9plus containing 0.06 µg/mL TGC. We use area

under the curve (AUC) to measure fitness of populations normalized by the AUC of their respective ancestor ($AUC_{evolved\ population}/AUC_{ancestor\ average}$). Analyses were done in RStudio (R v4.2.1) utilizing previously published pipelines (27) (https://github.com/mjfritz/Growth_Curves_in_R).

Quantitative reverse transcriptive PCR

To measure evolved efflux pump expression, we extracted RNA as described above for ancestors and day-12 TGC evolved populations grown in the presence (0.06 μg/mL TGC, ancestral cultures only) or absence of TGC (for ancestral and evolved populations). 50ng of purified RNA was added per reaction for Q-RT-PCR according to manufacturer's instructions for the Power SYBR Green RNA-to-Ct 1-Step Kit (Applied Biosystems). Reactions were performed in biological and technical triplicate and run on a QuantStudio3 thermocycler using the default threshold detection. Reactions lacking reverse transcriptase mix were included as controls for DNA contamination. Data were analyzed for ΔCt and ΔΔCt, normalized by *rpoB* and ancestor, respectively, within strain background. Expression values are presented as $2^{-normalized\ expression}$ such that higher values indicate increased expression. We used the main efflux pump gene (*adeB, G, and J)* as a representative for pump expression (28,29). Primers were designed with Geneious to amplify approximately 150 base pair regions and are listed in Table S4.

Ethidium bromide efflux activity assay

Efflux activity was measured with the ethidium bromide uptake assay loosely based on previously published methods (30). Ancestral cultures were grown overnight in M9plus, either with or without addition of 0.06 μg/mL TGC, but not until saturation, and 500 μL was pelleted, washed twice, and resuspended with PBS. Efflux activity of day-12 TGC-evolved populations was measured only in untreated M9plus. A black flat-bottom 96-well plate (Corning ref. 3925) was seeded with 90 μL of resuspended cells and 10 μL of 10 μg/mL ethidium bromide (Invitrogen by Thermo Fisher Scientific ref. 15585-011). Fluorescence intensity was measured at three minutes post addition of ethidium bromide with an excitation of 530 nm and emission of 600 nm. High efflux activity would result in a low fluorescence intensity as less ethidium bromide is left within the cells binding to DNA. We present efflux activity as 1/RFU such that higher values indicate more efflux. Efflux activity of the day-12 TGC-evolved populations when grown in the untreated state relative to respective ancestor was calculated as $1/(RFU_{query}/RFU_{untreated\ ancestor})$. Efflux activity was measured in biological triplicate with at least three technical replicates each.

SUPPLEMENTAL REFERENCES

1. Wijers CDM, Pham L, Menon S, Boyd KL, Noel HR, Skaar EP, et al. Identification of Two Variants of Acinetobacter baumannii Strain ATCC 17978 with Distinct Genotypes and Phenotypes. Infect Immun. 2021 Nov 16;89(12):e0045421.

2. Baumann P, Doudoroff M, Stanier RY. A study of the Moraxella group. II. Oxidative-negative species (genus Acinetobacter). J Bacteriol. 1968 May;95(5):1520–41.

3. Jacobs AC, Thompson MG, Black CC, Kessler JL, Clark LP, McQueary CN, et al. AB5075, a Highly Virulent Isolate of Acinetobacter baumannii, as a Model Strain for the Evaluation of Pathogenesis and Antimicrobial Treatments. MBio. 2014 May 27;5(3):e01076-14.

4. Gallagher LA, Ramage E, Weiss EJ, Radey M, Hayden HS, Held KG, et al. Resources for Genetic and Genomic Analysis of Emerging Pathogen Acinetobacter baumannii. J Bacteriol. 2015 Jun 15;197(12):2027–35.

5. Santos-Lopez A, Marshall CW, Scribner MR, Snyder DJ, Cooper VS. Evolutionary pathways to antibiotic resistance are dependent upon environmental structure and bacterial lifestyle. eLife. 2019 Sep 13;8.

6. Schwengers O, Jelonek L, Dieckmann MA, Beyvers S, Blom J, Goesmann A. Bakta: rapid and standardized annotation of bacterial genomes via alignment-free sequence identification. Microb Genom. 2021 Nov;7(11).

7. Tonkin-Hill G, MacAlasdair N, Ruis C, Weimann A, Horesh G, Lees JA, et al. Producing polished prokaryotic pangenomes with the Panaroo pipeline. Genome Biol. 2020 Jul 22;21(1):180.

8. Pritchard L, Glover RH, Humphris S, Elphinstone JG, Toth IK. Genomics and taxonomy in diagnostics for food security: soft-rotting enterobacterial plant pathogens. Anal Methods. 2016;8(1):12–24.

9. Jolley KA, Maiden MCJ. BIGSdb: Scalable analysis of bacterial genome variation at the population level. BMC Bioinformatics. 2010 Dec 10;11:595.

10. Feldgarden M, Brover V, Gonzalez-Escalona N, Frye JG, Haendiges J, Haft DH, et al. AMRFinderPlus and the Reference Gene Catalog facilitate examination of the genomic links among antimicrobial resistance, stress response, and virulence. Sci Rep. 2021 Jun 16;11(1):12728.

11. Tatusov RL, Galperin MY, Natale DA, Koonin EV. The COG database: a tool for genome-scale analysis of protein functions and evolution. Nucleic Acids Res. 2000 Jan 1;28(1):33–6.

12. CLSI C. M100: Performance Standards for Antimicrobial Susceptibility Testing. 32nd ed. Clinical and Laboratory Standards Institute; 2022.

13. Stephens AC, Thurlow LR, Richardson AR. Mechanisms Behind the Indirect Impact of Metabolic Regulators on Virulence Factor Production in Staphylococcus aureus. Microbiol Spectr. 2022 Aug 31;10(4):e0206322.

14. Bray NL, Pimentel H, Melsted P, Pachter L. Near-optimal probabilistic RNA-seq quantification. Nat Biotechnol. 2016 May;34(5):525–7.

15. Love MI, Huber W, Anders S. Moderated estimation of fold change and dispersion for RNA-seq data with DESeq2. Genome Biol. 2014;15(12):550.

16. Bliek T, Chouaref J, van der Kloet F, Szkodon J, Galland M. Introductionto RNA-seq lesson [Internet]. 2020 [cited 2024 Jul 1]. Available from: https://scienceparkstudygroup.github.io/rna-seq-lesson/

17. Zhu A, Ibrahim JG, Love MI. Heavy-tailed prior distributions for sequence count data: removing the noise and preserving large differences. Bioinformatics. 2019 Jun 1;35(12):2084–92.

18. Scribner MR, Santos-Lopez A, Marshall CW, Deitrick C, Cooper VS. Parallel Evolution of Tobramycin Resistance across Species and Environments. MBio. 2020 May 26;11(3).

19. Santos-Lopez A, Marshall CW, Haas AL, Turner C, Rasero J, Cooper VS. The roles of history, chance, and natural selection in the evolution of antibiotic resistance. eLife. 2021 Aug 25;10.

20. Huo W, Busch LM, Hernandez-Bird J, Hamami E, Marshall CW, Geisinger E, et al. Immunosuppression broadens evolutionary pathways to drug resistance and treatment failure during Acinetobacter baumannii pneumonia in mice. Nat Microbiol. 2022 Jun;7(6):796–809.

21. Travisano M, Mongold JA, Bennett AF, Lenski RE. Experimental tests of the roles of adaptation, chance, and history in evolution. Science. 1995 Jan 6;267(5194):87–90.

22. Santos-Lopez A, Fritz MJ, Lombardo JB, Burr AHP, Heinrich VA, Marshall CW, et al. Evolved resistance to a novel cationic peptide antibiotic requires high mutation supply. Evol Med Public Health. 2022 May 30;10(1):266–76.

23. Baym M, Kryazhimskiy S, Lieberman TD, Chung H, Desai MM, Kishony R. Inexpensive multiplexed library preparation for megabase-sized genomes. PLoS ONE. 2015 May 22;10(5):e0128036.

24. Turner CB, Marshall CW, Cooper VS. Parallel genetic adaptation across environments differing in mode of growth or resource availability. Evol Lett. 2018 Aug 4;2(4):355–67.

25. Deatherage DE, Barrick JE. Identification of mutations in laboratory-evolved microbes from next-generation sequencing data using breseq. Methods Mol Biol. 2014;1151:165–88.

26. Alonso-Del Valle A, León-Sampedro R, Rodríguez-Beltrán J, DelaFuente J, Hernández-García M, Ruiz-Garbajosa P, et al. Variability of plasmid fitness effects contributes to plasmid persistence in bacterial communities. Nat Commun. 2021 May 11;12(1):2653.

27. Muraski MJ, Nilsson EM, Fritz MJ, Richardson AR, Alexander RW, Cooper VS. Adaptation to Overflow Metabolism by Mutations That Impair tRNA Modification in Experimentally Evolved Bacteria. MBio. 2023 Apr 25;14(2):e0028723.

28. Coyne S, Courvalin P, Périchon B. Efflux-mediated antibiotic resistance in Acinetobacter spp. Antimicrob Agents Chemother. 2011 Mar;55(3):947–53.

29. Lin F, Xu Y, Chang Y, Liu C, Jia X, Ling B. Molecular Characterization of Reduced Susceptibility to Biocides in Clinical Isolates of Acinetobacter baumannii. Front Microbiol. 2017 Sep 26;8:1836.

30. Lonergan ZR, Nairn BL, Wang J, Hsu Y-P, Hesse LE, Beavers WN, et al. An Acinetobacter baumannii, Zinc-Regulated Peptidase Maintains Cell Wall Integrity during Immune-Mediated Nutrient Sequestration. Cell Rep. 2019 Feb 19;26(8):2009-2018.e6.
